# Construction of a Territorial Space Classification System Based on Spatiotemporal Heterogeneity of Land Use and Its Superior Territorial Space Functions and Their Dynamic Coupling: Case Study on Qionglai City of Sichuan Province, China

**DOI:** 10.3390/ijerph18179052

**Published:** 2021-08-27

**Authors:** Dinghua Ou, Qi Zhang, Yijie Wu, Jing Qin, Jianguo Xia, Ouping Deng, Xuesong Gao, Jinhu Bian, Shangqi Gong

**Affiliations:** 1 College of Resources, Sichuan Agricultural University, Chengdu 611130, China; oudinghua@hotmail.com (D.O.); zhangqi19970820@gmail.com (Q.Z.); wuyijay2218@163.com (Y.W.); qinjing0724@163.com (J.Q.); xiajianguo@126.com (J.X.); auh6@sicau.edu.cn (O.D.); gongsq@stu.sicau.edu.cn (S.G.); 2 Institute of Mountain Hazards and Environment, Chinese Academy of Sciences, Chengdu 610041, China; bianjinhu@imde.ac.cn

**Keywords:** territorial space classification, territorial space function, spatiotemporal heterogeneity, *q*-statistic, spatiotemporal analysis, spatial coupling

## Abstract

Territorial space classification (TSC) provides the basis for establishing systems of national territory spatial planning (NTSP) and supervising their implementation in China, thus has important theoretical and application significance. Most of the current TSC research is related to land use/land cover classification, ignoring the connection of the NTSP policies and systems, failing to consider the spatiotemporal heterogeneity of land use superior territorial space functions (TSFs) and the dynamic coupling between land use and its superior TSFs on the result of TSC. In this study, we integrated the factors influencing the connection of NTSP policies and systems and established a theoretical framework system of TSC from the perspective of spatial form and functional use. By integrating the q-statistic method with spatiotemporal geographical analysis, we propose a method to construct a TSC system for Qionglai City of Sichuan Province in China based on the spatiotemporal heterogeneity of land use superior TSFs and the dynamic coupling between land use and its superior TSFs. It makes up for the deficiency of directly taking land use/land cover classification as TSC and solves the problems of ignoring the spatiotemporal heterogeneity of land use superior TSFs and the dynamic coupling between land use and its superior TSFs. Using this method, we found that the TSC of Qionglai City consists of 3, 7, and 14 first-, second-, and third-level space types, respectively. The key findings from this study are that land use superior TSFs show spatiotemporal heterogeneity in Qionglai, and coupling effects in spatial distribution were noted between land use types and their superior TSFs, as was temporal heterogeneity in the coupling degree and the structure of the TSFs corresponding to the land use types, which show obvious dynamics and non-stationarity of the functional structure. These findings confirm the necessity of considering the spatiotemporal heterogeneity of land use superior TSFs and the dynamic coupling between land use and its superior TSFs in TSC. This method of establishing a TSC system can be used to address a number of NTSP and management issues, and three examples are provided here: (a) zoning of urban, agricultural, and ecological space; (b) use planning of production, living and ecological space; (c) delimitation of urban development boundary, permanent basic farmland protection redline, and ecological protection redline.

## 1. Introduction

Territorial space, i.e., the geographical space with national sovereignty, is the carrier and location of human production and life, providing the fundamental guarantee for economic and social development, including land and ocean territorial space [1]. Since the start of economic reform in 1978, China has witnessed rapid economic growth and urban expansion, resulting in a series of territorial space governance problems, such as inefficient use of resources, spatial mismatch of land–water resources, and ecological degradation [2,3]. Spatial planning is considered an important governance method for managing regional inconsistencies and unsustainable issues [4,5], aiming to optimize the layout of territorial space and enhance the capacity of spatial governance [1,6]. Regrettably, since the Communist Party of China took power in 1949, no national territory spatial planning (NTSP) system has been established for territorial space governance, which is considered to be more or less one of the main causes of the series of problems with China’s territorial space governance [7,8]. In recent years, to solve these issues and unsustainable development problems caused by the lack of spatial planning, China has adopted a number of reform measures (e.g., major institutional reforms, the unified spatial planning system, etc.) and is determined to restructure the existing spatial planning system to improve the national governance capacity [1]. Specifically, China’s Ministry of Natural Resources issued the *Guidelines for Classification of Land and Sea for Territorial Space Investigation, Planning, and Use Control (Trial)* in 2020 to better meet the needs of the formulation of an NTSP. However, this territorial space classification (TSC) system is faced with the same problems as the previous TSCs (e.g., the Second/Third National Land Survey Classification): they are two-dimensional (2D) space classification systems based on land use/land cover, without considering the territorial space function (TSF) and its spatiotemporal heterogeneity, creating barriers to territorial spatial zoning and use planning. The problem is not confined to China’s TSC; similar problems still exist in the widely used international TSC systems, such as in the United Nations Food and Agriculture Organization’s (FAO) land cover classification system [9], the European Community Collaboration for Environmental Information’s (CORINE) land cover classification system [10], the International Geosphere–Biosphere Program’s (IGBP) global land cover classification system [11], etc. The aim of this study is to reveal the complex spatiotemporal heterogeneity of the land use superior territorial space functions (TSFs) and the dynamic coupling between land use and its superior TSFs and propose a new method to construct a TSC system based on land use data to provide references for NTSP and management in China. 

### 1.1. Spatiotemporal Heterogeneity of Land Use Superior TSFs and Dynamic Coupling between Land Use and Its Superior TSFs

Land function refers to the capacity of the goods and services provided by a land system [12,13]. Not many international studies have used TSF as a specialized term. A few Chinese scholars have used TSF [14] and territorial space functional area and other related concepts in their research [15], but none of them provided a detailed definition. By comparing the connotation of territorial space [1] and land [16], it can be found that the meanings of territorial space and land are approximately the same in terms of nature and economy. For example, in China’s NTSP system [1], the three categories of national space, namely production, living, and ecology, are named according to their respective dominant functions. These dominant functions are essentially a broad generalization of the nine land use functions defined in SENSOR [17]. Moreover, some scholars have preliminarily divided land functions into production, living, and ecological functions [18,19,20]. Therefore, TSF is equated with land function in this study.

Multi-functionality is an essential attribute of land use [21,22]. The multi-functionality of land use is the result of the diversified use of land function (TSF), i.e., the extent to which the TSF provided by land use can meet the needs of human society [23,24]. Due to differences in the endowments of natural resources and socioeconomic conditions, the many TSFs provided by land use are necessarily different, but one or several TSFs invariably play a leading role. In this study, the predominant TSF provided by land use is defined as the land use superior (dominant) TSF. The superior TSF is not necessarily unique (it may be one or more), whereas the dominant TSF is theoretically unique, but the phenomenon of mixed dominant function, in reality, cannot be ruled out. For example, woodland may not only play a leading role in raw materials production function by providing wood and forest by-products, but it may also play a key role in climate regulation by regulating temperature (which is confirmed by the research results in this study). 

Land use and its functions have complex spatiotemporal heterogeneity [13,25,26,27]. In theory, their corresponding superior TSFs also have spatiotemporal heterogeneity, and these spatiotemporal heterogeneities inevitably affect TSC [13], which was the basic starting point of this research. Unfortunately, few studies have mentioned the dynamic coupling between land use and its superior TSFs (i.e., the coupling degree and its dynamics of the spatial heterogeneity of land use and its superior TSFs), and the impact of this dynamic coupling on the result of TSC, which was another starting point of this study and a major challenge faced in this research. 

### 1.2. The Problem of TSC Based on Land Use/Land Cover

According to different bases for classification, TSC research can be classified into single-perspective classification (e.g., population-density-based classification [28], land-cover-based classification [10], etc.) and multi-perspective classification (e.g., a combination of land-cover and population-density-based classifications [29], classifications based on a combination of publicly available data on resident and working population, CORINE land cover and infrastructure [30], etc.). Most of these TSCs are related to land use/land cover classifications. Existing classifications based on land use/land cover, however, suffer from at least one of the following drawbacks: 

First, land use/land cover classification is directly regarded as TSC, but the functional attributes of territorial space are ignored. For example, the FAO land cover classification system was developed based on the Anderson land cover classification system [31], and its classification is realized by defining some descriptive attributes of land cover, e.g., the forests are classified according to tree-canopy cover and tree height, not considering their superior (dominant) function [32]. Another example is the CORINE land cover classification system, which is mainly based on the geomorphic properties (shape, size, color, texture, and pattern) of landscape objects (natural, improved cultivation, and artificial) and the spatial relationship of landscape objects to classify land cover, but misjudgments may occur in the division of artificial surfaces that need to be identified with the help of functional attributes, such as rural residential areas shaded by tree canopies [10]. Classification methods of other common TSC systems, e.g., the land cover classification system of the IGBP [11], Anderson [31], and the United States Geological Survey (USGS) [33], are basically the same as the FAO and CORINE. They divide land types according to some attributes of land cover that can be easily extracted from remote sensing images and pay insufficient attention to land functions. Although these classification systems can represent the territorial space in 2D space, they cannot fully reflect their TSFs attributes and sufficiently connect with China’s territorial space governance policies (e.g., they cannot directly and quickly delimit the regulatory boundaries of production, living, and ecological space proposed in China’s NTSP system through land use data). Fortunately, the *Guidelines for Classification of Land and Sea for Territorial Space Investigation, Planning, and Use Control (Trial)* issued by China’s Ministry of Natural Resources in 2020 considers space for the implementation of the national ecological civilization construction system (e.g., the addition of wetlands), which provides a basis for the government’s ecological construction decision-making. However, the cohesion of China’s territorial space governance policies is still insufficient. Therefore, the need to develop a new method to construct a TSC system that is better adapted to the implementation of China’s current territorial space governance policies is urgent. 

Second, some TSC systems can be formulated by identifying and merging land use types on the basis of land-use classification. Most such methods of construction take the perspective of the dominant function of land use and establish connections and transformation relationships between land-use types and territorial space types through qualitative analysis. For example, Zhang et al. [34] summarized land use types and reconstructed a land classification system of NTSP for Hebi City through comprehensive comparative analysis. Zou et al. [35] identified and merged land use functions to construct an ecological–production–living TSC system by combining theoretical analysis and empirical research. These studies initially realized the cohesion and conversion of TSC and land use classification and provided the means for the application of statistical data, such as global land cover datasets and national land surveys data, as well as historical planning achievements such as land use planning, urban, and rural planning. However, these research methods were poor in quantitative analysis, restricting by the subjective factors related to qualitative analysis that introduced uncertainty in the level and priority of land use functions. 

Third, to solve these problems of qualitative analysis, some researchers have used quantitative methods to measure and identify land use functions to establish TSC systems. Zou et al. [36] and Liu et al. [37] quantitatively identified TSFs and constructed TSC systems using spatial models. Wandl et al. [30] developed a new TSC method by using a combination of publicly available data on the resident and working population, infrastructure, and CORINE land cover to realize the spatial classification of in-between territories and urban and rural areas. These studies enhance the theory of constructing TSC based on land use classification but ignore the spatiotemporal heterogeneity of land use superior TSFs and the dynamic coupling between land use and its superior TSFs. A few studies considered the spatial heterogeneity of land use [38] and the dynamics of classification indicators [39] but did not pay attention to the temporal heterogeneity of land use superior TSFs and the dynamic coupling between land use and its superior TSFs.

My goal in this study was to solve the problems in the current TSCs based on land use/land cover by proposing a new method of constructing a TSC system. To do this, we (a) constructed a theoretical framework of TSC by considering spatial form, functional use, and policy implementation; (b) quantified the TSFs and quantitatively identified the land use superior TSFs using functional values, mathematical models, and spatial interpolation; (c) established a method to identify the land use dominant TSFs based on the spatiotemporal heterogeneity of the land use superior TSFs and the dynamic coupling between land use and its superior TSFs by integrating the q-statistic in the geographical detector with geographic spatiotemporal analysis. We used this method to construct a county-level TSC system and relate it to the system of land use classification. The general intent of this work is to construct a TSC system of Qionglai City and demonstrate its connection with the land use classification system by proposing a new method of constructing a TSC system based on land use and providing an effective method to use the statistical data and planning results provided by the previous land classification system, as well as provide methodological reference and theoretical support for China’s NTSP (such as territorial spatial zoning and territorial use planning) and management (such as the delimitation of “three lines” of urban development boundary, permanent basic farmland protection redline, and ecological protection redline). 

## 2. Materials and Methods

### 2.1. Study Area and Data

#### 2.1.1. Study Area

**Figure 1:**
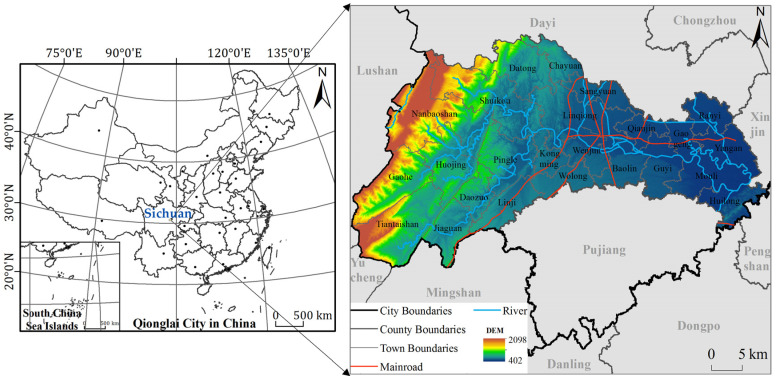
Geographical location of the study area.

Qionglai City is situated in the center of Sichuan Province, at 30°12′ N~30°33′ N and 103°04′ E~103°45′ E. It is located in the transition zone between the Chengdu Plain and Longmen Mountain and covers an area of 1377 km^2^, with 1 street, 19 towns, and 4 townships under its jurisdiction (Figure 1). The overall terrain decreases in altitude from the northwest to southeast, with the highest elevation of 1991 m and the lowest of 451 m. The area contains mountains, hills, and flatlands. To the east and northeast of the city are flatlands over an area of 311.36 km^2^. The mountainous area is 817.79 km^2^, with the Wumian and Changqiu Mountains in the south and the southern extension of Longmen Mountain in the west. The northwestern margin of the central area consists of deep hills over 245.98 km^2^. The river in the territory is 271.25 km long with abundant water resources. Qionglai City has a subtropical humid monsoon climate, with an average annual temperature of 16.3 °C, total rainfall of 1117.3 mm, 1107.9 h of sunshine, and 1024.92 mm of annual evaporation. The main soil types are aquic soil and purple soil. The vegetation features subtropical evergreen broad-leaved forests mainly distributed in the northwest low mountains and central hilly areas. Qionglai is the national new center of the western part of Tianfu New Area of Sichuan Province, with a focus on ecology and the tourism industry. In 2020, the city’s GDP was 33.073 billion yuan.

#### 2.1.2. Data Sources and Processing

The data used in this study mainly include raster data, vector data, sample monitoring data, and social-economic statistics. Details about the data used for analysis and their sources are shown in Table 1. As the data types were not unified and some monitoring data were missing, it was necessary to process the data and convert them into a consistent raster in the same coordinate system (the geographic coordinate system was WGS-84, and the projection coordinate system was UTM) at the same resolution (5 m) and in the same format (ESRI grid). The data that needed to be preprocessed included sample monitoring data, panel data, land use data, and raster data. (1) The sample monitoring data included the organic content of the soil, temperature, rainfall, and radiation. The organic content, monthly accumulated temperature higher than 10 °C, annual accumulated temperature higher than 10 °C, monthly rainfall, annual rainfall, and annual average rainfall was based on sample monitoring data using the Kriging interpolation method in ESRI ArcGIS 10.4 to obtain the corresponding raster surface. The maximum average standard errors of interpolation were 8.57 g/kg, 15.18 °C, 129.09 °C, 4.22 mm, 223.45 mm, and 160.12 mm, respectively, and met the requirements of data accuracy. Certain clarifications are in order. First, when the research project was launched, the official temperature and rainfall data for 2020 had not been released. The corresponding monitoring data for 2020 were thus predicted by applying a linear programming model on monitoring data from 2006 to 2019. Second, monitoring data on the organic content of the soil in the years 2015 and 2020 were lacking. Given that this content changes little in the short term in general [40], data on the organic content of the soil in 2016 and 2019 were used as monitoring data for its content in 2015 and 2020, respectively. (2) The panel data included social-economic data and were spatialized by the corresponding model and methods (details in Section 2.2.2) based on a 50 × 50 m spatial grid. Data for 2020 were predicted by a linear programming model based on data from 2001 to 2018. (3) Land use data for 2020 were obtained by using ESRI ArcGIS 10.4 to modify patches on the land use map in 2018 that have changed through referring to high-definition Google satellite images in 2020. Transportation networks, such as railways, highways, county roads, village roads, and farm roads, were identified by using images from Google and derived from the data of land use change surveys in the years of interest (Spatial distribution maps of land use types for 2010, 2015 and 2020 in Figure S1). (4) Data from the DEM, NDVI, remote sensing images, and raster images were transformed into raster data to meet the needs of research through coordinate transformation and resampling.

### 2.2. Research Methods

**Figure 2:**
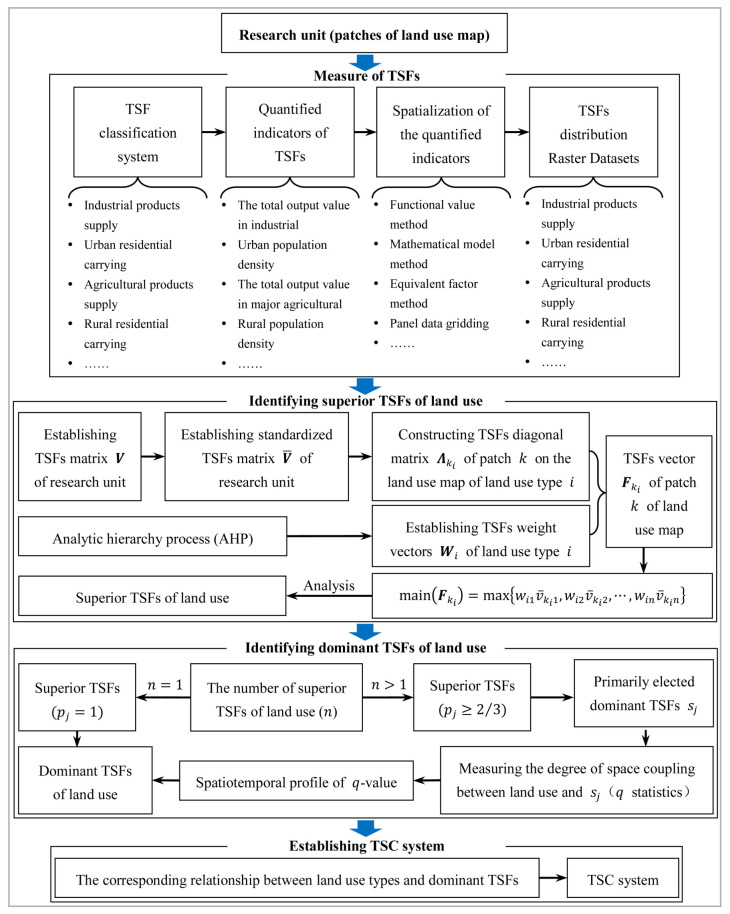
Diagram of the proposed framework. Note: The specific definition of $V,\bar{V},\Lambda_{k_{i}},W_{i},F_{k_{i}}$ is detailed in Equations (1)–(5), respectively; $p_{j}$ is the frequency of occurrence of the superior TSFs within the spatial scope of land use type i, $p_{j}=N/t$, where j is the territorial space type, N/t means that the superior TSFs corresponding to land use type i occurred *N* times in year *t*; q-value is the degree of space coupling between land use and $s_{j}$, which is defined in the Equation (6).

Land use is a physical form of expression of territorial space. Due to differences in resources, the suitability of land for various uses, and the diversity of social needs, the same kind of land use can accommodate different TSFs, resulting in a complex interaction between them. On the one hand, the same kind of land use entities can realize different TSFs through different methods of utilization (e.g., woodland can serve the raw materials production function by providing wood and forest by-products, or serve as an aesthetic draw for tourists); on the other hand, the same TSFs can be realized in different land use entities (e.g., natural ecological functions can be realized to varying degrees by forest lands, grasslands, and water bodies). Therefore, to clarify the dominant TSFs among land use entities, we need to classify territorial space and quantify and identify the superior TSFs. Then, based on the spatiotemporal heterogeneity of these superior TSFs in the context of land use, we need to identify the dominant TSFs to establish the corresponding relationship between land use types and TSC. This can help realize “nonobjective” TSFs expressed through “real” land use, provide a link between statistical data and the results of planning of land classification systems, and can increase the sources of available data for territorial space planning. The research framework is shown in Figure 2.

#### 2.2.1. TSC

The suitability of territorial resources for multiple purposes determines that the given space can be used for different ends, which shows that the space of the same region has multiple functions. It is precisely because of the versatility of territorial space that TSC is the basis for the functional quantification of space. We construct a theoretical framework with three levels of classification of territorial space (Table 2)—three first-level types, eight second-level types, and 16 third-level types (Description of the third-level types of territorial space classification in Table S1)—by combining the developmental goals of the “three basic spaces” (production, living, and ecological), proposed in a report at the 18th National Congress of the Communist Party of China (CPC), with requirements of the demarcation of three zones (urban, agricultural, and ecological), proposed in the *Opinions on the Establishment and Supervision of the Implementation of the Territorial Space Planning System* by the CPC’s Central Committee and the State Council. We also comprehensively consider the actual situation of county territorial space planning and the availability of data, as well as integrate the form of TSF and human development demand, and refer to the relevant theoretical research [17,35,36,37,41,42,43,44,45,46] and technical specifications, such as the *National Ecological Function Zoning (Revised Edition)*, *Guidelines for Delineation of Ecological Protection Red Lines*, and *Municipal Territorial Space Master Planning Compilation Guide*. The first level of classification of the system focuses on the requirements of delineation of the three zones and divides the entire territorial space into three major parts—urban, rural, and natural ecological spaces—according to the form of use of space. Classification at the second level follows the concept of the “three basic spaces” [35,36,37] and refines the first-level classification from the perspective of spatial function. Urban space is subdivided into spaces for urban production and urban living, rural space is divided into spaces for rural production and rural living, and the natural ecological space is divided into spaces for supply services and regulation services, support services, and cultural services. The third-level classification is based on the use of space and refined the second-level classification to obtain quantifiable functional service areas.

Urban space is territorial space used for production and living by urban residents [47], thus, forming its dominant function. From the perspective of functional attributes, urban space is divided into spaces for urban production and living. Urban production space has the function of supplying industrial products and commercial services or auxiliary production within the scope of urban space. As industrial products and commercial services are its main functions [36], it can be further divided into areas for the supply of industrial products and service industrial products according to the use of space. Urban living space is the projection of various types of activities and social relationships that constitute people’s daily lives within the scope of urban space. Employment, travel, medical care, education, ecological security, and other guarantees of life and housing are the basic functional uses of urban living space [35,48]. It is further divided into areas of urban residential carrying and urban living security. 

Rural space is a region with large areas of agricultural or forestry-related land use or a large amount of uncultivated land, including small human settlements dominated by agricultural production [49]. Similar to the division of urban space, we divide rural space into spaces for rural production and living according to their functional attributes. The space for rural production is made up of the three basic elements of “background of production activity, infrastructure network, and rural complex” [50]. The rural complex is an ideological space, whereas the functional spaces considered here are objectively existing material spaces. Starting from the background of production activity and the infrastructure network, we divide the rural production space into areas for the supply of agricultural products and transportation services. Rural living space is a multi-level regional complex of living, employment, leisure, social interaction, consumption, and public service activities by rural residents [51]. The residential function is the foundation of rural living space, and such public service functions as employment, medical care, and education provide the main support for the rural living space [51]. Therefore, rural living space is divided into areas with rural residential carrying and living security. 

Natural ecological space is territorial space with natural attributes and the dominant function of providing ecological products or ecological services [42]. By referring to the Millennium Ecosystem Assessment (MA) and authoritative research results [42,43,45,46], we divided the natural ecological space into spaces for supply services, regulation services, support services, and cultural services from the perspective of functional attributes. To meet the requirements of controlling the use of space, the space for the supply services was subdivided into areas for raw materials production and water supply, the space for regulation services was subdivided into areas for gas regulation, climate regulation, and environmental purification, the space for support services was subdivided into areas of soil conservation and biodiversity maintenance, and the space for cultural services was specified as the area of aesthetic landscape.

#### 2.2.2. Quantifying TSF and Identifying Land Use Superior TSFs

(1) Determining quantitative indicators of TSFs and its spatialization methods: This study selected quantitative indicators of TSFs according to principles including clear indicator connotation, simple quantification method, and easy data acquisition, and then we applied methods of functional measurements and spatialization (e.g., functional value method, equivalent factor method, spatial interpolation, and panel data gridding, etc.) to spatialize them [42,52,53,54,55] (Table 3). Finally, we obtained the spatially distributed raster data of the functional indicators and used them to establish the TSFs matrix of patches on the land use map by ESRI ArcGIS 10.4:

(1) $$V={\left(v_{kj}\right)}_{l\times n}\;\;\left(k=1,2,\cdots ,l;j=1,2,\cdots n\right)$$

where $v_{kj}$ is the original function value of territorial space type j on patch k of the land use map, l is the number of patches of the land type of land use map, and n is the number of TSF types. 

The threshold method was used for the dimensionless processing of the TSFs to eliminate the interference of dimensionality on the comparison of TSF sizes. A standardized TSF matrix $\bar{V}$ of patches of the land use map was thus obtained.

(2) $$\bar{V}={\left({\bar{v}}_{kj}\right)}_{l\times n}\;\;\left(k=1,2,\cdots ,l;j=1,2,\cdots n\right)$$

where ${\bar{v}}_{kj}$ is the standardized function value of the territorial space type j on patch k, and its standardization method is ${\bar{v}}_{k.}=\frac{\max\left(v_{k.}\right)-v_{k.}}{\max\left(v_{k.}\right)-\min\left(v_{k.}\right)}$, ${\bar{v}}_{k.}$ is the standardized value of a specific TSF of the k-th patch of the land use map. 

We then establish the standardized diagonal matrix of TSF for patch k of land use type i:

(3) $$\Lambda_{k_{i}}=\mathrm{diag}\left({\bar{v}}_{k_{i}1},{\bar{v}}_{k_{i}2},\cdots ,{\bar{v}}_{k_{i}n}\right)$$

where ${\bar{v}}_{k_{i}j}\left(j=1,2,\cdots ,n\right)$ is the standardized function value of territorial space type j in the k-th patch of land use type i.

(2) Determining the responses of TSFs for land use type i: The same land use entity can realize different TSFs through different methods. However, in terms of functional use, the responses of the same land use type to different TSFs are bound to be different. Therefore, the weights of responses of land use type to different TSFs are determined by using the analytic hierarchy process (AHP) [56], and weight vectors of the TSFs for land use type i are obtained:

(4) $$W_{i}=\left[w_{i1},w_{i2},\cdots ,w_{in}\right]\;\;\left(i=1,2,\cdots ,m\right)$$

where $w_{ij}$ is the weight of the j-th TSF corresponding to land use type i. 

(3) Determining the superior TSF for the k-th patch of land use type i: The identification of land use superior TSFs is to find out the dominant function among various TSFs corresponding to the given land use types from the perspective of the overall function of territorial space, so as to determine the superior TSF for a specific patch on the land use map. Based on this, the TSF vector $F_{k_{i}}$ of patch k is calculated according to Formula (5). Then, the TSF corresponding to the maximum function value $\max\left\{w_{i1}{\bar{v}}_{k_{i}1},w_{i2}{\bar{v}}_{k_{i}2},\cdots ,w_{in}{\bar{v}}_{k_{i}n}\right\}$ in $F_{k_{i}}$ is regarded as the superior TSF of patch k of land use type i:

(5) $$F_{k_{i}}=W_{i}\Lambda_{k_{i}}$$

**Table 3:** Quantified indicators and spatial methods of the TSFs.

Code	Name	Indicators	Formula	Explanation of Parameters
U11	Industrial products supply	The total output value in industrial	$Pv_{i}=\frac{GIP\times A_{i}}{A}$	$Pv_{i}$ is the total output value in industrial of grid i; GIP is the total output value in industrial of Qionglai; A is the total area of mining and industrial land in Qionglai; $A_{i}$ is the total area of mining and industrial land in grid i.
U12	Service industrial products supply	The total output value in the tertiary industry (wholesale-retail, accommodation-catering, and real estate)	$Sv_{i}=\frac{n_{i}\times \left(Wv+Av+Rv\right)}{N}$	$Sv_{i}$ is the total output value in the tertiary industry (wholesale-retail, accommodation-catering, and real estate) of grid i; Wv, Av, and Rv are the output value of wholesale-retail, accommodation-catering and real estate respectively; $n_{i}$ is the total number of POI interest points in wholesale and retail, accommodation and catering, real estate industry in grid i; N is the total number of POI interest points of wholesale-retail, accommodation-catering, and real estate in Qionglai.
U21	Urban residential carrying	Urban population density	When the grid is all located within the boundaries of the town: $Cd_{i}=\frac{c_{i}\times Cp_{i}}{C_{i}\times s_{net}}$ When the grid is divided into n partitions by the town:$Cd_{i}=\frac{1}{S_{net}\sum_{j=1}^{n}c_{j}}\left(\frac{c_{1}^{2}\times Cp_{1}}{C_{1}}+\frac{c_{2}^{2}\times Cp_{2}}{C_{2}}+\cdots +\frac{c_{n}^{2}\times Cp_{n}}{C_{n}}\right)$	$Cd_{i}$ is the population density of the town in grid i; $c_{i}$ and $C_{i}$ are, respectively, the land area of urban and organic town of grid i and the town where grid i is located; $Cp_{i}$ is the urban population of the town where grid i is located; $s_{net}$ is the grid area; $c_{1}$, $c_{2},$ and $c_{n}$ are, respectively, the land areas of town and organic town in the 1st, 2nd, and n-th partitions of grid i; $C_{1}$, $C_{2},$ and $C_{n}$ are, respectively, the land areas of town and organic town of the towns where the 1st, 2nd, and n-th partitions are located in grid i; $Cp_{1}$, $Cp_{2},$ and $Cp_{n}$ are, respectively, the urban population of the towns in the 1st, 2nd, and n-th partitions of grid i.
U22	Urban living security	The public service capacity index in urban	$CI_{i}=\frac{1}{4}Er_{i}+\frac{1}{4}Sr_{i}+\frac{1}{4}Mr_{i}+\frac{1}{4}Or_{i}$ $Er_{i}=\frac{CP_{i}\times Ce}{CP\times CP}$ $Sr_{i}=S_{1i}/S_{1}$ $Mr_{i}=S_{2i}/S_{2}+S_{3i}/S_{3}$ $Or_{i}=S_{4i}/S_{4}$	$CI_{i}$ is the urban public service capability index of grid i; $Er_{i}$ is the ratio of urban employed population in grid i; $CP_{i}$ is the urban population of grid i; Ce is the total number of urban employees in Qionglai; CP is the urban population of Qionglai; $Sr_{i}$, $Mr_{i},$ and $Or_{i}$ are, respectively, the proportion of research and education land, medical and health land, and other living security land in grid i; $S_{1i}$, $S_{2i}$, $S_{3i}$, and $S_{4i}$ are, respectively, the areas of research and education land, medical and health land, the number of POI points of interest in clinics and pharmacies, and the area of specially designated land of grid i; $S_{1}$, $S_{2}$, $S_{3}$, and $S_{4}$ are, respectively, the total area of research and education land, the total area of medical and health land, the total number of POI points of interest in clinics and pharmacies, and the total area of specially designated land in Qionglai.
R11	Agricultural products supply	The total output value in major agricultural	When the grid is all located within the boundaries of the town: $Av_{i}=F\times \frac{A_{i}}{A}$ When the grid is divided into n partitions by the town:$Av_{i}=\sum_{j=1}^{n}\frac{a_{j}\times Av_{j}}{A_{j}}$	$Av_{i}$ is the total output value in major agriculture in grid i; F is the total output value in major agriculture of the town; $A_{i}$ is the total area of cropland, forest land, grassland, and water body in grid i; A is the total area of cropland, forest land, grassland, and water body in the town; $a_{j}$ is the total area of cropland, forest land, grassland, and water body in the j-th partition of grid i; $A_{j}$ is the total area of cropland, forest land, grassland, and water body of the town where the j-th partition of grid i is located; $Av_{j}$ is the total output value in major agriculture of the town where the j-th partition of grid i is located.
R12	Transportation services supply	Road network density	$D_{i}=\sum_{i=1}^{6}w_{i}R_{i}$	$D_{i}$ is the road network density of grid i; $w_{i}$ is the weight of road type i (the weights of railway, expressway, national/provincial road, county/township road, village road, and farm road are set to 0.27, 0.23, 0.20, 0.16, 0.13, 0.01, respectively); $R_{i}$ is the road density of type i in grid i.
R21	Rural residential carrying	Rural population density	When the grid is all located within the boundaries of the town: $Vd_{i}=\frac{v_{i}\times Vp_{i}}{V_{i}\times S_{net}}$ When the grid is divided into n partitions by the town: $Vd_{i}=\frac{1}{S_{net}\sum_{j=1}^{n}v_{j}}\left(\frac{v_{1}^{2}\times Vp_{1}}{V_{1}}+\frac{v_{2}^{2}\times Vp_{2}}{V_{2}}+\cdots +\frac{v_{n}^{2}\times Vp_{n}}{V_{n}}\right)$	$Vd_{i}$ is the rural population density of grid i; $v_{i}$ and $V_{i}$ are, respectively, the village land area of grid i and the town where grid i is located; $Vp_{i}$ is the rural population of the town where grid i is located; $s_{net}$ is the grid area; $v_{1}$, $v_{2,}$ and $v_{n}$ are, respectively, the village land area of the 1st, 2nd, and n-th partitions of grid i; $V_{1}$, $V_{2,}$ and $V_{n}$ are, respectively, the village land area of the towns where the 1st, 2nd, and n-th partitions are located in grid i; $Vp_{1}$, $Vp_{2},$ and $Vp_{n}$ are, respectively, the rural population of the towns in the 1st, 2nd, and n-th partitions of grid i.
R22	Rural living security	The public service capacity index in rural	$VI_{i}=\frac{1}{4}Ar_{i}+\frac{1}{4}Sr_{i}+\frac{1}{4}Mr_{i}+\frac{1}{4}Or_{i}$ $Ar_{i}=\frac{VP_{i}\times Ae}{VP\times VP}$ $Sr_{i}=S_{1i}/S_{1}$ $Mr_{i}=S_{2i}/S_{2}+S_{3i}/S_{3}$ $Or_{i}=S_{4i}/S_{4}$	$VI_{i}$ is the rural public service capability index of grid i; $Ar_{i}$ is the employment rate of agricultural population in grid i; $VP_{i}$ is the number of rural population in grid i; Ae is the total number of agricultural employees in Qionglai; VP is the total rural population of Qionglai; $Sr_{i}$, $Mr_{i}$, $Or_{i}$, $S_{1i}$, $S_{2i}$, $S_{3i}$, $S_{4i}$, $S_{1}$, $S_{2}$, $S_{3},$ and $S_{4}$ have the meanings stated in the formula of $CI_{i}$.
E11	Raw materials production	Net primary productivity of vegetation	Improved CASA model	See references for details [57].
E12	Water supply	Water conservation	$Q_{i}=\sum_{j=1}^{n}\left(P_{i}-R_{ij}-ET_{i}\right)\times A_{ij}$ $R_{ij}={\bar{P}}_{i}\times \alpha_{ij}$	$Q_{i}$ is the water resources conservation amount of grid i; $P_{i}$ is the annual rainfall of grid i; $R_{ij}$ is the annual surface runoff of ecosystem type j of grid i; $ET_{i}$ is annual evapotranspiration of grid i; $A_{ij}$ is the area of ecosystem type j of grid i; j is the serial number for ecosystem types; n is the number of ecosystem types of grid; ${\bar{P}}_{i}$ is the annual average rainfall of grid i; $\alpha_{ij}$ is the average surface runoff coefficient of the ecosystem type j of grid i, and these values can be obtained by the “*Guidelines for Delineation of Ecological Protection Red Lines*”.
E21	Gas regulation	Carbon fixation and oxygen release	$C_{i}=NPP_{i}\times \beta \times N_{C}+NPP_{i}\times \delta$	$C_{i}$ is the amount of carbon fixation and oxygen release in grid i; $NPP_{i}$ is net primary productivity of vegetation in grid i; $N_{C}$ is the content of C in CO_2_, and its value is 27.27%; β and δ are 1.63 and 1.19, respectively, indicating that 1.63 g CO_2_ and 1.19 g O^2^ were absorbed and fixed by plants for every 1 g dry matter (biomass) production.
E22	Climate regulation	Evapotranspiration	$ET_{i}=\frac{463\times RMI_{i}}{1+e^{-\left[1.79\Sigma_{i}+1.34\right]}}+150$ $\Sigma_{i}=\sum_{j=1}^{12}NDVI_{ij}{}_{\left(NDVI_{ij}>0.05\sum {\bar{T}}_{ij}>0{}^{\circ}C\right)}$ $RMI_{i}=MI_{i}/{\bar{MI}}_{i}$ $MI_{i}=\frac{P_{i}}{0.16\sum {\left(T_{ij}\right)}_{T\geq 10{}^{\circ}C}}$	$ET_{i}$ is annual evapotranspiration of grid i; $RMI_{i}$ is the relative humidity index of grid i; $NDVI_{ij}$ is the j-th month average normalized difference vegetation index of grid i; $\Sigma_{i}$ is the cumulative value of $NDVI_{ij}$ greater than 0.05 in the period when the monthly average temperature ${\bar{T}}_{ij}$ is greater than 0 in grid i; $MI_{i}$ is the moisture index of grid i; ${\bar{MI}}_{i}$ is the annual average moisture index of grid i; $P_{i}$ is the annual precipitation of grid i; $\sum {\left(T_{ij}\right)}_{T\geq 10{}^{\circ}C}$ is the cumulative value of accumulated temperature greater than 10 °C in one year.
E23	Environmental purification	Equivalence of environmental purification service	$EV_{i}=\sum_{k=1}^{m}Ev_{k}s_{ik}$	$EV_{i}$ is equivalent to the environmental purification service of grid i; $Ev_{k}$ is equivalent of environmental purification service per unit area of ecosystem type k; $s_{ik}$ is the area of ecosystem type k in grid i; m is the number of ecosystem types.
E31	Soil conservation	Soil conservation	RUSLE	See references for details [58].
E32	Biodiversity maintenance	Habitat quality	INVEST	See references for details [59].
E41	Aesthetic landscape	Equivalence of aesthetic landscape service	$AV_{i}=\sum_{k=1}^{m}Av_{k}s_{ik}$	$AV_{i}$ is equivalent to the aesthetic landscape service of grid i; $Av_{k}$ is equivalent of aesthetic landscape service per unit area of ecosystem type k; $s_{ik},$ and m have the meanings stated in the formula of $EV_{i}$.

#### 2.2.3. Spatiotemporal Heterogeneity of Land Use TSFs and Identification of Dominant TSFs

There is spatiotemporal heterogeneity in land use TSFs, i.e., there are differences in the TSFs of the same land use type in different regions, and the TSF corresponding to the land use in the same area changes over time. This leads to differences in the TSFs of the same land use type in different regions changes over time. Obviously, the same land use type within a certain spatial scope is bound to have multiple superior TSFs. Identifying the dominant TSF from among these superior TSFs is difficult using only their values without considering the influence of spatiotemporal heterogeneity. The degree of coupling between land use and the spatial heterogeneity of its superior TSFs over many years (2010, 2015, 2020) was detected through q-statistic method [60,61], and using the spatiotemporal profile of coupling degree to portray the dynamics of their spatial heterogeneity [62]. We then identified the dominant TSF by comprehensive analysis (note: when only one same superior TSF was identified for a certain type of land use over many years, it was directly identified as the dominant TSF for that land use type). 

(1) Primary election of dominant TSF of land use type i: The number and structure of superior TSFs in terms of land use also change over time owing to the spatiotemporal heterogeneity of TSFs. Assuming that the superior TSFs $s_{j}\left(j=1,2,\cdots ,n\right)$ corresponding to land use type i occurred N times in year t, their frequency of occurrence within the spatial scope of land use type i is $p_{j}=N/t$. Superior TSFs with $p_{j}\geq 2/3$ were extracted as the primarily elected dominant TSFs of land use type i. 

(2) Coupling of spatial distribution between land use type i and its primarily elected dominant TSF: The coupling of spatial distribution between land use type i and its primarily elected dominant TSF $s_{j}$ was measured by using q-statistic method [60], and the specific steps are as follows:

Step 1: We used the natural breakpoint method in ESRI ArcGIS 10.4 to divide the patches of primarily elected dominant TSF $s_{j}$ in the land use type i into class L based on the standardized values ${\bar{v}}_{kj}$ of the TSFs.
Step 2: We counted the number N of all patches on the land use map and the number $N_{h}$ of patches belonging to class h for the primarily elected dominant TSF $s_{j}$ of land-use type i. We then calculated the mean $\bar{Y}=\left(1/N\right)\sum_{i=1}^{N}Y_{i}$, $\mu_{h}=\left(1/N_{h}\right)\sum_{i=1}^{N_{h}}Y_{hi}$, and variance $\sigma^{2}=\left(1/N\right)\sum_{i=1}^{N}{\left(Y_{i}-\bar{Y}\right)}^{2}$, $\sigma_{h}^{2}=\left(1/N_{h}\right)\sum_{i=1}^{N_{h}}{\left(Y_{hi}-\mu_{h}\right)}^{2}$ of the areas of all patches on the land use map and patches belonging to class h, where $Y_{i}$ and $Y_{hi}$ are the areas of all patches i and patches i in class h of the primarily elected dominant TSF $s_{j}$ of land-use type i, respectively.
Step 3: The q-value in the q-statistic method was calculated using the formula [60]:
  (6) $$q=1-\frac{\sum_{h=1}^{L}N_{h}\sigma_{h}^{2}}{N\sigma^{2}}$$
  where h is the type code of the primarily elected dominant TSFs $s_{j}$, h=1,2,⋯,L, and q is the degree of coupling of spatial distribution between land use type i and its primarily elected dominant TSFs $s_{j}$, indicating that the primarily elected dominant TSFs $s_{j}$ explained 100×q% of land use type i. Its value was in the range [0, 1]; a larger reflected a stronger explanatory power of the primarily elected dominant TSFs $s_{j}$ for land use type i (a higher degree of coupling). In particular, q=1 meant that the primarily elected dominant TSFs $s_{j}$ was fully coupled with their corresponding land use in the spatial distribution, and q=0 meant that the primarily elected dominant TSFs $s_{j}$ was not associated with the corresponding land use.
Step 4: The significance of the q-value was tested as follows: First, we calculated the F-value and λ according to Formulae (7) [60] and (8) [60], respectively. Then, applied Keisan online calculation service [https://keisan.casio.com/ (accessed on 9 February 2020)] to calculate $F_{\alpha }\left(L-1,\;N-L,\lambda \right)$ and p-value. Finally, we determined the significance of the q-value, i.e., when $\;F>F_{\alpha }\left(L-1,N-L,\lambda \right)$ and p<α, q was statistically significant, indicating that the coupling of spatial distribution between land use type i and its primarily elected dominant TSFs $s_{j}$ was significant at the level α:
  (7) $$F=\frac{N-L}{L-1}\frac{q}{1-q}$$
  (8) $$\lambda =\frac{1}{\sigma^{2}}\left[\sum_{h=1}^{L}\mu_{h}^{2}-\frac{1}{N}{\left(\sum_{h=1}^{L}\sqrt{N_{h}}\mu_{h}\right)}^{2}\right]$$

(3) Identifying the dominant TSFs of land use type i: Owing to the spatiotemporal heterogeneity of land use TSFs, the degree of coupling (q-value) between land use type i and its primarily elected dominant TSFs $\;s_{j}$ also exhibited spatiotemporal heterogeneity, and it was difficult to identify the dominant TSFs using only the value of q. We visualized the degree of coupling between land use and its primarily elected dominant TSFs by drawing the spatiotemporal profile of q to visually represent the pattern of dynamic changes in it and identified the dominant TSF corresponding to land use type i by comprehensive analysis.

## 3. Results and Analysis

### 3.1. Analyzing Spatiotemporal Heterogeneity of Superior TSFs of Land Use

**Figure 3:**
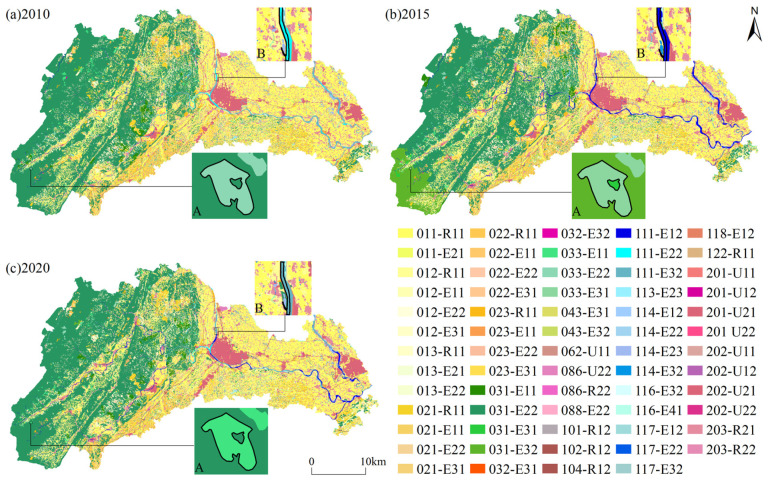
Spatial distribution of the land use superior TSFs in Qionglai. (**a**–**c**) are spatial distribution of the land use superior TSFs in 2010, 2015 and 2020. R11, E11 and so on have the meanings stated in Table 2; 011, 012 and so on are the land class code, have the meanings stated in the land use status classification in China (GB/T 21010-2007); e.g., 102-R11 indicates that the superior TSF of paddy field is agricultural products supply.

From 2010 to 2020, a general spatiotemporal heterogeneity in the superior TSFs was noted corresponding to the land use types in Qionglai (Figure 3). It was mainly manifested as follows (sparsely forested woodland and river are used as examples):

(1) The superior TSFs corresponding to the same land use type in the same location changed over time. From 2010 to 2020, the corresponding superior TSFs of sparsely forested woodland consisted of the functions of climate regulation, raw materials production, and soil conservation but changed significantly in the same location over time. Taking patch A as an example (Figure 3), the superior TSF in 2010 was climate regulation but changed to soil conservation in 2015 and raw materials production in 2020. From 2010 to 2020, the superior TSFs corresponding to river were climate regulation, biodiversity maintenance, and water supply and exhibited strong temporal heterogeneity at the same location. Taking patch B as an example (Figure 3), its superior TSF was climate regulation in 2010, water supply in 2015, and biodiversity maintenance in 2020. Similarly, there was significant temporal heterogeneity in the superior TSFs corresponding to the same land type in the same location for other land use types (Figure 3).
(2) Differences were observed in the spatial distribution of superior TSFs corresponding to the same land use type at the same time. We use sparsely forested woodland as an example. In 2010, the superior TSF of sparsely forested woodland in the northern part was raw materials production, while that in the southern part was climate regulation. The superior TSF was soil conservation in the central and southwestern parts of sparsely forested woodland. In the context of river, the eastern part of the river in 2010 mainly served to maintain biodiversity, water supply was scattered in its western part, and its central part regulated the climate. The spatial distribution of superior TSFs on the same land use type thus varied significantly. Similarly, the superior TSFs corresponding to the same land use type in other land use types in different periods also showed complex spatial heterogeneity (Figure 3).
(3) The spatial distribution of superior TSFs corresponding to the same land use type and differences between them changed over time. We consider sparsely forested woodland as an example. From 2010 to 2020, the spatial distribution of raw materials production gradually shifted from the north to the south for this land use type, that of climate regulation shifted from the south to the north, and the spatial distribution of soil conservation tended to become scattered from a concentrated distribution (central and southwest). Taking river as another example, during 2010–2020, the spatial distribution of water supply shifted from the west to the east in river, biodiversity maintenance changed from being concentrated in the east to being scattered in the west of the river, and gradually returned to the east. Climate regulation first changed from being concentrated in the middle of river to being scattered along its edges and then returned to the middle for this land use type. Similarly, the spatial distribution of superior TSFs corresponding to other land use types also exhibited prominent and complex spatiotemporal heterogeneity (Figure 3).

### 3.2. Analyzing Spatiotemporal Coupling between Land Use and Superior TSFs

There are 26 land use types in the study area, but only 14 land use types, such as paddy field, irrigated cropland, and rainfed cropland, were analyzed in this study for the spatiotemporal coupling of their superior TSFs (Table 4). This is mainly because survey data of rural land use change used in this study did not divide city, organic town, village, and specially designated land, and thus could not distinguish the internal TSFs of these land use types. In addition, only one superior TSF was identified for mining land, land for scenic site facilities, land for agricultural facilities, highway, rural road, railway, hydraulic structure, and reservoir. Thus, a spatiotemporal coupling analysis of the superior TSF was not carried out on these land use types.

(1) A coupling was noted between the layout of land use and the spatial distribution of its superior TSFs. Each land use type had at least one superior TSF, and the spatial distribution of the superior TSF is coupled with it (i.e., q was significant) (Table 4). Four land use types—canal and ditch, inland mudflat, other grasslands, and other orchards—were coupled only with the spatial distribution of one superior TSF each—water supply, biodiversity maintenance, soil conservation, and agriculture products supply, respectively. The remaining 10 types of land use were all coupled with the spatial distributions of multiple superior TSFs. In most periods, orchards and croplands were coupled with the spatial distribution of two superior TSFs, and both were significantly coupled with the spatial distribution of the supply of agricultural products. More superior TSFs were coupled in terms of spatial distribution with forest lands and water bodies than with orchards and croplands. For example, a coupling was found between woodland, and raw materials production, climate regulation, biodiversity maintenance, soil conservation. A coupling was also found between river and climate regulation, biodiversity maintenance, and water supply. In general, all land use types had superior TSFs coupled to them; most were coupled to multiple superior TSFs, while only a few were coupled to one superior TSF.
(2) Temporal heterogeneity was observed in the coupling between land use layout and the spatial distribution of its superior TSFs. On the one hand, the superior TSFs coupled with land use were unstable, and differed in different periods (Table 4). For example, in 2010 and 2020, tea plantation was coupled only with one kind of functional spatial distribution, of agricultural products supply, but in 2015 was coupled with the supply of agricultural products, as well as climate regulation. In 2015 and 2020, pond was coupled with the spatial distributions of the four functions of climate regulation, biodiversity maintenance, environmental purification, and water supply, whereas, in 2010, it was coupled with climate regulation, biodiversity maintenance, and environmental purification. On the other hand, the degree of coupling between land use type and superior TSFs was unstable and changed with time. For instance, the coupling (q-value) between river and its superior TSFs increased first and then decreased over time; the coupling between pond, inland mudflat, sparsely forested woodland, woodland, and the superior TSFs of most natural ecological spaces decreased first and then increased. This might have occurred because Qionglai is exemplary of the idea of the construction of a national ecological civilization and the main area of the “*Chengdu ‘Western Control’ Strategic Master Plan (2017–2035)*”. In recent years, ecological construction has been implemented under this project to control developmental intensity, promote green and low-carbon industries, and gradually restore and improve regional ecological functions. Therefore, the coupling between these land use types and the superior TSFs of most natural ecological spaces increased.

### 3.3. Analyzing the Results of Identification of Land Use Dominant TSFs

**Figure 4:**
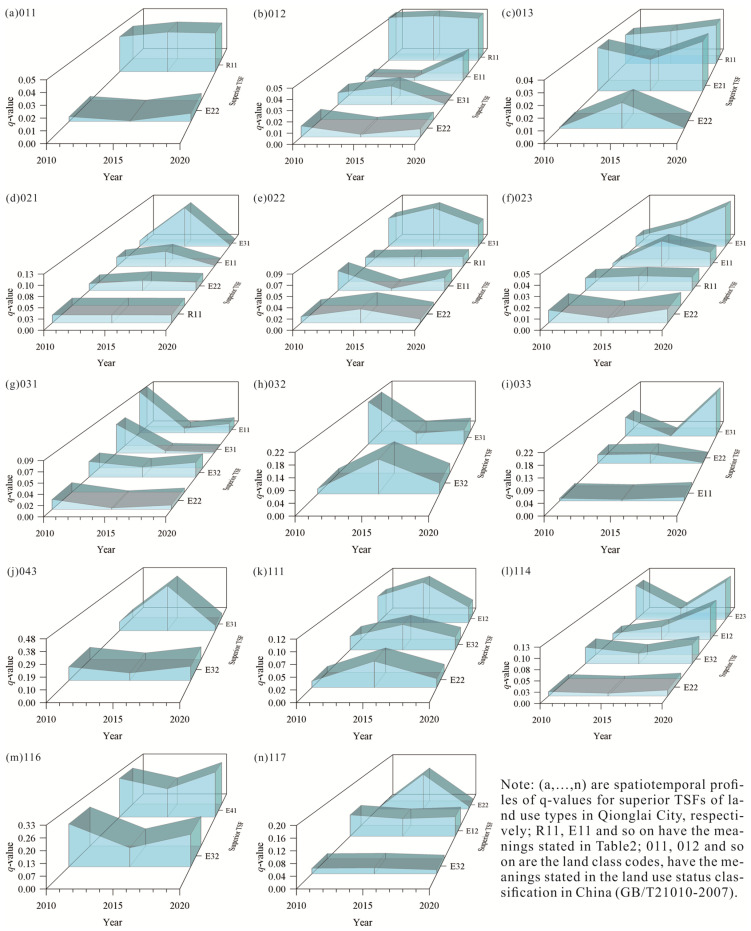
Spatiotemporal profiles of q-values for superior TSFs of land use types in Qionglai City during 2010–2020.

In the study area, the structure and degree of coupling of TSFs with the land use layout exhibited temporal heterogeneity, so the dominant land use TSF could not be determined simply based on the q-value. For example, superior TSFs coupled with fruit plantation had the largest q-value for raw materials production in 2015, but the q-values of this function were not significant in 2010 and 2020, because of which it was clearly not reasonable to consider it as the dominant TSF of this land use type. Thus, we identified dominant TSFs by analyzing the spatiotemporal profiles of the q-values (Figure 4).

(1) The dominant TSFs of croplands. The croplands in Qionglai City are of three types: paddy field, rainfed cropland, and irrigated cropland. The degree of coupling (q-value) of these three types with the supply of agricultural products was greater than that of other superior TSFs in the same land use type. The q-value of the supply of agricultural products fluctuated by little (the standard deviation σ is 0.0021, 0.0037, 0.0012, respectively) and which the significance was high, and thus, was chosen as the dominant TSF for these three land use types.
(2) The dominant TSFs of orchards. Orchards in Qionglai City are of three types: tea plantation, fruit plantation, and other orchards. The q-values of soil conservation were the largest in the three years among superior TSFs corresponding to tea plantation, which were 0.06128, 0.08374, 0.0472, respectively, but were not statistically significant at all levels considered. The q-values of climate regulation and raw materials production were highly variable over time and unstable (σ is 0.0070, 0.0133, respectively). Although the q-values of supply of agricultural products were smaller than the maximum value in the three years considered, the overall fluctuation was small (σ is 0.0006), and its q-values were stable and significant at the level of α=0.001. It was, thus, regarded as the dominant TSF. Similarly, the supply of agricultural products was regarded as the dominant TSF of other orchards. The corresponding climate regulation and supply of agricultural products of fruit plantation had significant q-values over the three years, but the q-value of climate regulation was less stable than that of the supply of agricultural products (The former σ is 0.0036, the latter σ is 0.0009). The latter was thus taken as the dominant TSF of fruit plantation.
(3) The dominant TSFs of forest land. Qionglai City has three types of forest land: woodland, shrubbery land, and sparsely forested woodland. Among the superior TSFs corresponding to woodland, the q-values of soil conservation were only significant in 2010, indicating that this function was poorly coupled with the spatial distribution of woodland. The q-value of raw materials production was significant for the three years. Its q-value in 2010 was 0.08924, which was the maximum for each superior TSFs in the three years. The q-value of biodiversity maintenance fluctuated slightly in the range 0.01862–0.02865 and was highly stable (σ is 0.0044). Although the q-values of climate regulation were smaller than the maximum value in the three years considered, they were significant in all three years at the level of α=0.001, indicating a high coupling of the spatial distribution of this superior TSFs with woodland. Therefore, raw materials production, biodiversity maintenance, and climate regulation were considered the dominant TSFs of woodland. Among the superior TSFs corresponding to shrubbery land, the q-values of soil conservation were significant in 2010 and 2020, while those of biodiversity maintenance were significant only in 2015. Soil conservation was thus taken as the dominant TSF of shrubbery land. Among the superior TSFs corresponding to sparsely forested woodland, the q-values of climate regulation fluctuated widely (σ is 0.0173), and were not significant in 2020; although the q-values of soil conservation were much greater than those for other functions in 2010 and 2020, they were significant only in 2020; as the q-values of raw materials production showed some volatility, the overall variation was smooth (σ is 0.0036) and its q-values were significant in all three years. Raw materials production was thus selected as the dominant TSF of sparsely forested woodland.
(4) The dominant TSF of grassland. Qionglai has only one type of grassland: other grasslands. Among its corresponding superior TSFs, biodiversity maintenance had insignificant q-values in all three years while soil conservation had an insignificant q-value for one year, but its q-values for the other two years were significant at the level of α=0.05. It was thus taken as the dominant TSF of other grasslands.
(5) The dominant TSFs of water bodies and water conservations facilities. Qionglai City has four types of water bodies and water conservations facilities: river, pond, inland mudflat, and canal and ditch. Among the superior TSFs corresponding to river, climate regulation had the smallest q-value in the three years, which were 0.01278, 0.05277, 0.01753, respectively, and its q-values fluctuated widely over time (σ is 0.0178), and they were significant only in 2015. The three-year q-values of both water supply and biodiversity maintenance were significant, but those of the former were the largest in the three years, which were 0.07324, 0.11152, 0.04284, respectively. It was thus taken as the dominant TSF of river. The q-values of the four superior TSFs corresponding to pond showed large fluctuations, among which the q-values of the water supply had the fastest growth rate but poor stability (σ is 0.0328), and was not significant in 2010. Thus, it is not suitable to be taken as the dominant TSF. Although the q-values of climate regulation, biodiversity maintenance, and environmental purification were significant in the three years, the environmental purification had the highest spatial coupling with pond in two of the three years (2010 and 2020), which q-values were 0.10676, 0.11936, respectively, and was thus taken as the dominant TSF of pond. Over the three years, both inland mudflat and canal and ditch were coupled with the spatial distribution of one superior TSF each, biodiversity maintenance and water supply, respectively. They were thus selected as dominant TSFs for these land use types.
(6) The dominant TSFs of land for cities, organic towns, villages, mining, and industries. It was not possible to identify the corresponding dominant TSFs of city, organic town, village, and specially designated land because the research data with the classification of rural land use survey in the land use status classification in China(GB/T21010-2007) did not subdivide these land use types. Thus, all superior TSFs that occurred more than two-thirds of the time in the three years were chosen as the dominant TSFs for these land use types, i.e., the dominant TSFs for city and organic town were the supply of industrial products and industrial service products, urban residential carrying, and urban living security; those for specially designated land were urban living security and rural living security, and those for village were rural residential carrying and rural living security. For the eight land use types of highway, rural road, railway, mining land, land for agricultural facilities, hydraulic structure, reservoir, and land for scenic site facilities, only one superior TSF each was identified for the three years, i.e., industrial products supply, climate regulation, agricultural products supply, water supply, and environmental purification were chosen as the dominant TSF of mining land, land for scenic site facilities, land for agricultural facilities, hydraulic structure and reservoir, respectively. The supply of transportation services was taken as the dominant TSF of highway, rural road, and railway.

### 3.4. Results of the TSC System

We established the corresponding relationships between land use types and territorial space types according to the dominant TSFs of each land use type (Figure 4), and so obtained the TSC of Qionglai and its corresponding land use types (Table 5). 

The TSC system of Qionglai included three first-level types, seven second-level types, and 14 third-level types. Compared with the theoretical framework of TSC (Table 2) proposed earlier in the study, there was no space for cultural services in the second-level territorial space and no areas of gas regulation and aesthetic landscapes in the third-level territorial space of Qionglai. This is because the degree of coupling and stability of gas regulation and aesthetic landscape, along with their corresponding land use layouts, were not as good as those of the other functions. It shows that the spatiotemporal heterogeneity of land use superior TSFs and the dynamic coupling of land use and its superior TSFs affect the results of TSC. Therefore, the method based on the spatiotemporal heterogeneity of land use superior TSFs and the dynamic coupling of land use and its superior TSFs is beneficial for constructing the TSC system. It can make the constructed TSC system more in line with the actuality of the interest area and avoid the deviations caused by using qualitative analysis to construct the theoretical system, thus promoting the further improvement of the NTSP level.

## 4. Discussion 

### 4.1. Beneficial Contributions of the Proposed Classification 

The TSC system and method addresses three problems related to the current TSCs based on land use/land cover classification, such as not considering the cohesion of national policies, the subjective limitation of using qualitative analysis methods to identify the territorial space type, and ignoring the spatiotemporal heterogeneity of land use superior TSFs and the dynamic coupling between land use and its superior TSFs in the quantitative identification of territorial space types. The proposed classification system and method are beneficial because they can complement the NTSP and management in China and other similar regions in the world, such as land use planning and supervision system formulation, territorial space zoning, and county-level NTSP. 

First, international far-reaching TSCs (e.g., FAO [9], CORINE [10], etc.) and the latest classification of China’s territorial industry (e.g., the Third National Land Survey Classification, and *Guidelines for Classification of Land and Sea for Territorial Space Investigation, Planning, and Use Control*) are mainly based on the descriptive characteristics of land cover. Although these TSCs can completely cover the territorial space on a 2D plane, which cannot express the functional attributes of territorial space and meet the needs of spatial planning to express composite territorial space. Especially, these classification systems do not consider the cohesive space of China’s NTSP policies and systems such as the “three zones and three lines” and “three basic spaces”, creating challenges for NTSP and management decision-making. From the perspective of spatial form and functional use, we propose a theoretical framework system of county-level TSC with three major spaces (urban, rural, and natural ecological), three types of functions (production, living, and ecological), and multiple functional uses as the core, which not only inherits the advantages of land use/land cover classification but also considers the functional attributes and use of territorial space. Moreover, it also fully combines the requirements of China’s national policies and systems such as “three zones and three lines” and “three basic spaces”, which compensates for the deficiency of directly taking land use/land cover classification as the TSC. Compared with the above TSCs, the advantages of the proposed classification system and method are: (a) The types of territorial space are identified, and a TSC system is established based on land use classification, which can demonstrate the connection and transformation between TSC and land use classification. Thus, we can take advantage of the land use classification, i.e., it can establish a corresponding relationship with most industry-related space classifications in China [34,41], so as to absorb and utilize the historical data results formed by the spatial classification of land, construction, and planning departments, and provide strong data support for the compilation of NTSP. (b) The spatial distribution and quantitative characteristics of urban, rural, and ecological spaces in the area of interest are directly obtained based on the land use data and the first-level types of the TSC system constructed in this study (Figure S2), which provides a reference for the delimitation and the formulation of control measures of “three zones and three lines”. (c) The production-living-ecological spaces are determined by simply merging the second-level types of the TSC system constructed in this study (i.e., the production space is obtained by merging urban and rural production spaces, the living space is obtained by merging the urban and rural living spaces, and the ecological space is obtained by merging the supply services, regulation services, and support services spaces). This provides a basis for formulating the “three basic spaces” development objectives and implementing the NTSP and management system.

Second, compared with the proposed method, previous researches on the construction of TSC systems by using qualitative methods have some problems such as insufficient quantitative analysis and low credibility. For example, some researchers used qualitative analysis methods (e.g., subsumption-based classification [34], geographic ontologies [63], and so on) to identify territorial space types based on land use classification, which were theoretically limited by the subjective factors of qualitative analysis, such as the lack of mathematical indicators for the determination of function size and priority, and the low credibility of the results obtained using only prior knowledge judgment. As a result, the reliability of the classification system is low, and the practical applications are limited. The proposed method of constructing a TSC system uses the patch of the land use as the identification unit to quantitatively identify the dominant TSF of land use through the measurement of TSF, establishes the corresponding relationship between territorial space type and land use type, and compensates for the subjective limitation of qualitative analysis. It considerably enhances the reliability of the connection and transformation between the land use types and the territorial space types and improves the implementation and generalization of the TSC system. For example, using the proposed method to construct the TSC system of Qionglai City, the corresponding relationships between all levels and types of territorial spaces and land use types in Qionglai City were established. Based on these relationships, the land use data can be used to generate the corresponding territorial space type distribution map more conveniently (Figures S2 and S3), which considerably improves the acceptability of the TSC system and enhances its applicability.

Third, in recent years, researchers have realized the limitations of qualitative analysis in constructing TSC systems. Some researchers tried to construct a TSF evaluation index system to quantitatively identify territorial space types and establish TSC systems [19,36,37]. Unfortunately, almost all of them ignored the spatiotemporal heterogeneity of land use superior TSFs and the dynamic coupling between land use and its superior TSFs. Especially, the superior TSF corresponding to the land use type has spatiotemporal heterogeneity, and the spatial coupling degree between land use and its superior TSF has obvious temporal heterogeneity, as well as obvious non-stationary characteristics of the superior TSF structure coupled with it (confirmed in Section 3.1 and Section 3.2). To solve this problem, we proposed a new method of constructing the TSC: (a) First, the coupling degree of spatial distribution between each land use type and its superior TSFs was measured by using the q-statistic method to quantitatively portray the spatial heterogeneity of land use superior TSFs. (b) Using this, the spatiotemporal profile of the q-values of the superior TSFs of each land use type was drawn over three years to dynamically depict the dynamic characteristics and temporal heterogeneity of the structure of the superior TSFs. (c) Finally, the land use dominant TSF was determined through the comprehensive analysis of the spatiotemporal profile of the q-values to establish the TSC system and its corresponding relationship with land use classification system. According to the relevant literature, this study is the first to integrate the spatiotemporal heterogeneity of land use superior TSFs and the dynamic coupling between land use and its superior TSFs into the construction of the TSC system, which provides a new idea and direction for the construction of TSC systems. To a large extent, the TSC system constructed by the proposed method in the study can overcome the problem caused by the possible deviation of territorial zoning based on the current functional status in the compilation of China’s NTSP, and improve the planners’ awareness of the spatiotemporal heterogeneity of TSFs, thereby promoting the scientificity and rationality of China’s NTSP, especially the county-level NTSP.

### 4.2. Limitations and Improvements of the Study

Although some problems existing in the current TSC have been addressed, some limitations and deficiencies must be noted, for example, the measurement of the differences of the impact of land use types on different TSFs, how to solve the non-one-to-one relationship between land use types and territorial space types in practical applications, the proposed method of constructing TSC systems requires many basic data and complex processing, etc. In this section, these deficiencies are discussed in detail, and some possible improvements are suggested.

First, when applying the AHP to determine the response weights of land use types to different TSFs, the subjective limitation of the judgment matrix relying on experts’ knowledge and experience was compensated for by inviting multiple experts (three experts in land, three experts in ecology, three experts in agriculture, three experts in planning, and three experts in economics) to determine the judgment matrix independent of each other, and repeatedly communicating with the experts to ensure that the judgment matrix fully passed the logical rigor test. However, the formulation process of this judgment matrix is too complicated, and if the expert provides improper choices or the method operation steps are nonstandard, the response weight measurement result is likely to be affected by subjective factors, and measurement results of the TSFs corresponding to land use types will deviate from reality to some extent. Subjective weighting methods (e.g., AHP, optimal sequence diagram method, etc.) and objective weighting methods (e.g., factor analysis method, entropy weight method, etc.) can be combined to determine weight to ensure the accuracy of the response weight and reduce the impact of subjective misjudgment in future research.

Second, there was a limitation whereby one land use type corresponded to multiple types of territorial space in the TSC system in this study. On the one hand, because the classification of rural land use survey in the land use status classification in China (GB/T21010-2007) did not subdivide the construction land such as city, organic town, and village, and thus could not distinguish among their corresponding TSFs within the given land use types, which is not convenient to quickly determine the TSFs by using the land use data, to a certain extent. These land use types can be subdivided by referring to high-resolution images, and the corresponding TSFs can then be identified according to the proposed method based on the subdivided land use type. On the other hand, due to the non-unique and non-exclusive nature of the land use dominant TSFs [36,64], there are a few land use types corresponding to multiple types of territorial space among the non-constructive land, which brings difficulties to the division of main functions (dominant functions) of territorial space based on land use type data. In applications, the main function of territorial space within land use types can be divided according to the functional benefit per unit area or the spatiotemporal heterogeneity of the TSFs. For example, although woodland corresponds to the three major functions of raw materials production, climate regulation, and biodiversity maintenance, due to the influence of the spatiotemporal heterogeneity of the TSFs, the three functions corresponding to woodland can be distinguished in space (Figure 3), which can meet the needs of functional pattern reconstruction in the formulation of NTSP. 

Third, to realize the construction method of the TSC system proposed in this study, 16 functional quantitative indicators need to be calculated for at least three years, and it needs to obtain and process a large amount of basic data such as land use, remote sensing images, ecological environment, and social economy. There are some problems, such as difficult data acquisition and heavy workload of calculation and processing, which will hinder the promotion and application of this method in practice to a large extent. In the next step, based on the proposed method, professional software will be developed for the TSC system integrating data acquisition, processing, calculation, analysis, output, and other functions to solve the above-mentioned problems in the application of this method and improve its replicability. In addition, the study area, Qionglai City, covers most land types in China (there are 38 secondary classifications in the Second China Rural Territorial Survey Classification, and Qionglai City covers 26 land use types). However, due to the geographical location and physical geography condition, the study area does not contain land use/land cover types such as glaciers and permanent snow, desert, and sea, etc. We expect more researchers to apply the proposed method to conduct research and applications of constructing TSC systems in other regions with different land use/land cover to further test and improve the proposed method in this study.

## 5. Conclusions 

The aim of this study was to construct a unified and scientific TSC system through comprehensive considering spatial form, functional use, and policy implementation. A method of constructing this TSC system based on the spatiotemporal heterogeneity of land use superior TSFs and the dynamic coupling between land use and its superior TSFs was innovatively proposed by integrating the q-statistic and geographic spatiotemporal analysis. As a case study, the TSC system of Qionglai was successfully constructed that demonstrated its connection with the land use classification system.

There are some obvious deficiencies in the current application and research of TSC based on the land use/land cover type. Some researchers directly treated land use/land cover classification as the TSC, ignoring the functional attributes of territorial space, whereas most land use/land cover classifications simply failed to consider the cohesion space with the NTSP policies and systems. Some studies lacked quantitative analysis, the established connection and conversion relationship between land use types and territorial space types were low in credibility, and the application prospects are poor. In the few quantitative studies of TSC based on land use classification, the spatiotemporal heterogeneity of the land use superior TSFs and the dynamic coupling between land use and its superior TSFs were hardly considered. In particular, this study confirms that the superior TSF corresponding to the land use type has spatiotemporal heterogeneity, and the spatial coupling degree between the land use and its corresponding superior TSF also has obvious temporal heterogeneity, and the structural non-stationary characteristics of its coupled superior TSFs are obvious.

The proposed method of constructing a TSC system solves the above problems, and the feasibility of the method was proven by constructing the TSC system of Qionglai City. The results confirm that: (a) A general spatiotemporal heterogeneity was noted in the superior TSFs corresponding to the land use types in Qionglai, indicating that it is necessary to consider the spatiotemporal heterogeneity of land use superior TSFs in the construction of TSC systems. (b) A coupling was noted between the land use pattern and the spatial distribution of its superior TSFs. Both the degree of coupling and the structure of the TSFs coupled with it show temporal heterogeneity, significant dynamics, and non-stationarity. The territorial space type corresponding to land use cannot be determined by the size of the q-value alone but by the dominant TSF determined by a comprehensive analysis of the spatiotemporal heterogeneity of land use superior TSFs and the dynamic coupling between land use and its superior TSFs with the help of spatiotemporal profiles. (c) The TSC system of Qionglai City consists of 3, 7, and 14 first-, second-, and third-level types of spaces, respectively. Compared with the theoretical TSC framework system (Table 2), there was no space for cultural services in the second-level territorial space and no gas regulation and aesthetic landscapes in the third-level territorial space of Qionglai City. This indicates that the spatiotemporal heterogeneity of land use superior TSFs and the dynamic coupling between land use and its superior TSFs affect the results of TSC. 

In conclusion, we argue that the established TSC system and its construction method have three main advantages: (a) The theoretical framework of TSC was established from the perspective of spatial form and functional use considering the implementation of national policies. The TSC system compensates for the deficiency directly regarding land use/land cover classification as the TSC and enriches the theoretical system of TSCs. (b) Based on considering patches of the land use map as units and function measurements as links, a method was proposed to quantitatively identify the territorial space types based on land use types and to construct relationships between the TSC and land use types. This helps avoid the deviations caused by using qualitative analysis to construct the TSC system and enhances the reliability of TSC results. (c) With the help of the q-statistic method and spatiotemporal profile, this paper overcomes the problems caused by ignoring the spatiotemporal heterogeneity of land use superior TSFs and the dynamic coupling between land use and its superior TSFs in the quantitative identification of territorial space types based on land use types, which provides a new method of TSC. 

## Figures and Tables

**Table 1:** Data sources and descriptions.

Data Type	Data Name	Time-Series (Year)	Data Accuracy	Data Source
Raster data	DEM	2020	12.5 m	91 Visitor Assistant
	Google satellite image	2020	0.51 m	91 Visitor Assistant
	NDVI	2010–2020	500 m	USGS
Vector data	Administrative boundary	2010, 2015, 2020	1:5000	Sichuan Academy of Land Science and Technology
	Land use status data	2010, 2015, 2018	1:5000	Sichuan Academy of Land Science and Technology
	Digital maps	2010, 2015, 2020	1:10,000	Geographical Information Monitoring Cloud Platform, 91 Visitor Assistant
Sample monitoring data	Soil organic matter content	2010	1271 sample points	Soil testing and fertilization project in Qionglai and adjacent counties
		2016	521 sample points	Cropland quality grade evaluation project in Qionglai and adjacent counties
		2019	921 sample points	Cropland quality monitoring and evaluation project for rotation and fallow areas in Qionglai and adjacent counties
	Soil particle composition	2010–2020	1 km	Geographical Information Monitoring Cloud Platform
	Temperature, rainfall	2006–2019	County-level (62 stations)	Resource and Environment Science and Data Center
	Radiation	2010, 2015, 2019	County-level	Meteorological Science Knowledge Service System
Social-economic statistics	The total output value in industrial, tertiary industry (wholesale-retail, accommodation-catering, and real estate), major agricultural, urban and rural population, agriculture, and urban employees	2001–2018	The total output value in industrial, tertiary industry (wholesale-retail, accommodation-catering, and real estate), major agricultural, and urban employees are county-level panel data; the urban and rural population and the agricultural employees are town level panel data	Qionglai Statistics Bureau

**Table 2:** Theoretical framework of TSC.

First-Level Types		Second-Level Types		Third-Level Types	
Code	Name	Code	Name	Code	Name
U	Urban space	U1	Urban production space	U11	Industrial products supply
				U12	Service industrial products supply
		U2	Urban living space	U21	Urban residential carrying
				U22	Urban living security
R	Rural space	R1	Rural production space	R11	Agricultural products supply
				R12	Transportation services supply
		R2	Rural living space	R21	Rural residential carrying
				R22	Rural living security
E	Natural ecological space	E1	Supply services space	E11	Raw materials production
				E12	Water supply
		E2	Regulation services space	E21	Gas regulation
				E22	Climate regulation
				E23	Environmental purification
		E3	Support services space	E31	Soil conservation
				E32	Biodiversity maintenance
		E4	Cultural services space	E41	Aesthetic landscape

**Table 4:** Consistencies in spatial distribution between land use types and their superior TSFs in 2010, 2015, and 2020.

Year	Paddy Field		Irrigated Cropland				Rainfed Cropland		
	R11	E22	E11	R11	E22	E31	R11	E22	E22
2010	0.03833 ***	0.00411 **	0.00452	0.05191 ***	0.01011 ***	0.01221	0.02652 ***	0.00082	0.03431
2015	0.04331 ***	0.00053	0.00322	0.05391 ***	0.00246	0.01898	0.03005 ***	0.01727	0.02498
2020	0.04217 ***	0.00693 ***	0.02742 ***	0.05091 ***	0.00735 ***	0.001	0.03553 ***	0.00038	0.03933 **
**Year**	**Fruit Plantation**				**Tea Plantation**				**Other Orchards**
	**E22**	**E11**	**R11**	**E31**	**R11**	**E22**	**E11**	**E31**	**E11**
2010	0.01831 ***	0.02714	0.01888 ***	0.01786	0.01830 ***	0.01045	0.03612	0.06128	0.00376
2015	0.02719 ***	0.04387 ***	0.01904 ***	0.12108	0.01933 ***	0.02274 **	0.00382	0.08373	0.02826
2020	0.02229 ***	0.0032	0.01872 ***	0.0054	0.01977 ***	0.00613	0.02372	0.0472	0.01575
**Year**	**Other Orchards (Continued)**			**Woodland**				**Shrubbery Land**	
	**R11**	**E22**	**E31**	**E11**	**E22**	**E32**	**E31**	**E32**	**E31**
2010	0.01283 ***	0.01164	0.01186	0.08924 ***	0.01658 ***	0.02865 ***	0.05779 ***	0.02018	0.20750 *
2015	0.01503 ***	0.00448	0.02558	0.01012 **	0.00257 ***	0.01862 **	0.00469	0.13396 *	0.05353
2020	0.01376 ***	0.01269	0.04837	0.02000 **	0.00719 ***	0.02692 **	0.00246	0.04142	0.06980 **
**Year**	**Sparsely Forested Woodland**			**Other Grasslands**		**River**			**Pond**
	**E11**	**E22**	**E31**	**E32**	**E31**	**E22**	**E32**	**E12**	**E22**
2010	0.00961 **	0.03746 ***	0.09476	0.11254	0.09361 **	0.01278	0.03240 **	0.07324 **	0.01052 ***
2015	0.00523 *	0.04157 ***	0.00149	0.06182	0.47216 **	0.05277 ***	0.06054 **	0.11152 ***	0.00661 **
2020	0.01404 **	0.00296	0.21576 **	0.11639	0.05381	0.01753	0.03019 **	0.04284 **	0.01410 ***
**Year**	**Pond (Continued)**			**Inland Mudflat**		**Canal and Ditch**			
	**E32**	**E23**	**E12**	**E32**	**E41**	**E22**	**E32**	**E12**	
2010	0.04346 ***	0.10676 ***	0.01789	0.24319 **	0.27946	0.00239	0.01852	0.08503 **	
2015	0.02766 ***	0.03362 **	0.03781 ***	0.10736 *	0.20385	0.15937	0.01987	0.07019 ***	
2020	0.04996 ***	0.11936 ***	0.09522 ***	0.18385 *	0.32367	0.01209	0.01333	0.07748 ***	

Note: R11, E11 and so on have the meanings stated in Table 2; “*, **, ***” means that the q-value was significant at the level of 0.1, 0.05, and 0.001, respectively. The q-value hierarchical statistical information for 2010, 2015, and 2020 in Tables S2–S4.

**Table 5:** TSC system in the city of Qionglai.

First-Level Types		Second-Level Types		Third-Level Types		Land Use Types
Code	Name	Code	Name	Code	Name	Name of Land Use Type (Code)
U	Urban space	U1	Urban production space	U11	Industrial products supply	City (201), organic town (202), mining land (204)
				U12	Service industrial products supply	City (201), organic town (202)
		U2	Urban living space	U21	Urban residential carrying	City (201), organic town (202)
				U22	Urban living security	City (201), organic town (202), specially designated land (205)
R	Rural space	R1	Rural production space	R11	Agricultural products supply	Paddy field (011), rainfed cropland (013), irrigated cropland (012), fruit plantation (021), tea plantation (022), other orchards (023), land for agricultural facilities (122)
				R12	Transportation service supply	Railway (101), highway (102), rural road (104)
		R2	Rural living space	R21	Rural residential carrying	Village (203)
				R22	Rural living security	Village (203), specially designated land (205)
E	Natural ecological space	E1	Supply services space	E11	Raw materials production	Woodland (031), sparsely forested woodland (033)
				E12	Water supply	River (111), canal and ditch (117), hydraulic structure (118)
		E2	Regulation services space	E22	Climate regulation	Woodland (031), land for scenic site facilities (205)
				E23	Environmental purification	Reservoir (113), pond (114)
		E3	Support services space	E31	Soil conservation	Shrubbery land (032), other grasslands (043)
				E32	Biodiversity maintenance	Woodland (031), inland mudflat (116)

## Data Availability

The data presented in this study are available on request from the corresponding author.

## Supplementary Materials

The following are available online at https://www.mdpi.com/article/10.3390/ijerph18179052/s1, Figure S1. Spatial distribution of land use types in Qionglai. (a–c) are spatial distribution map of land use types in 2010, 2015 and 2020. Figure S2. Spatial distribution of the first-level types of territorial space classification in Qionglai. (a–c) are spatial distribution map of the first-level types of territorial space classification in 2010, 2015 and 2020. Figure S3. Spatial distribution of the second-level types of territorial space classification in Qionglai. (a–c) are spatial distribution map of the second-level types of territorial space classification in 2010, 2015 and 2020. Table S1. Description of the third-level types of territorial space classification in Table 2. Table S2. The q-value hierarchical statistical information table in 2010. Table S3. The q-value hierarchical statistical information table in 2015. Table S4. The q-value hierarchical statistical information table in 2020.
