# Supplementary material for: Construction of a Territorial Space Classification System Based on Spatiotemporal Heterogeneity of Land Use and Its Superior Territorial Space Functions and Their Dynamic Coupling: Case Study on Qionglai City of Sichuan Province, China
Source: Int J Environ Res Public Health. 2021 Aug 27;18(17):9052. doi: 10.3390/ijerph18179052 (PMC8431555; doi:10.3390/ijerph18179052)
Supplement: Supplementary file 1 [file ijerph-18-09052-s001.zip › ijerph-1329506-supplementary.pdf]

Figure S1. Spatial distribution of land use types in Qionglai. (a, b, c) are spatial distribution map of land use types in 2010, 2015 and 2020.

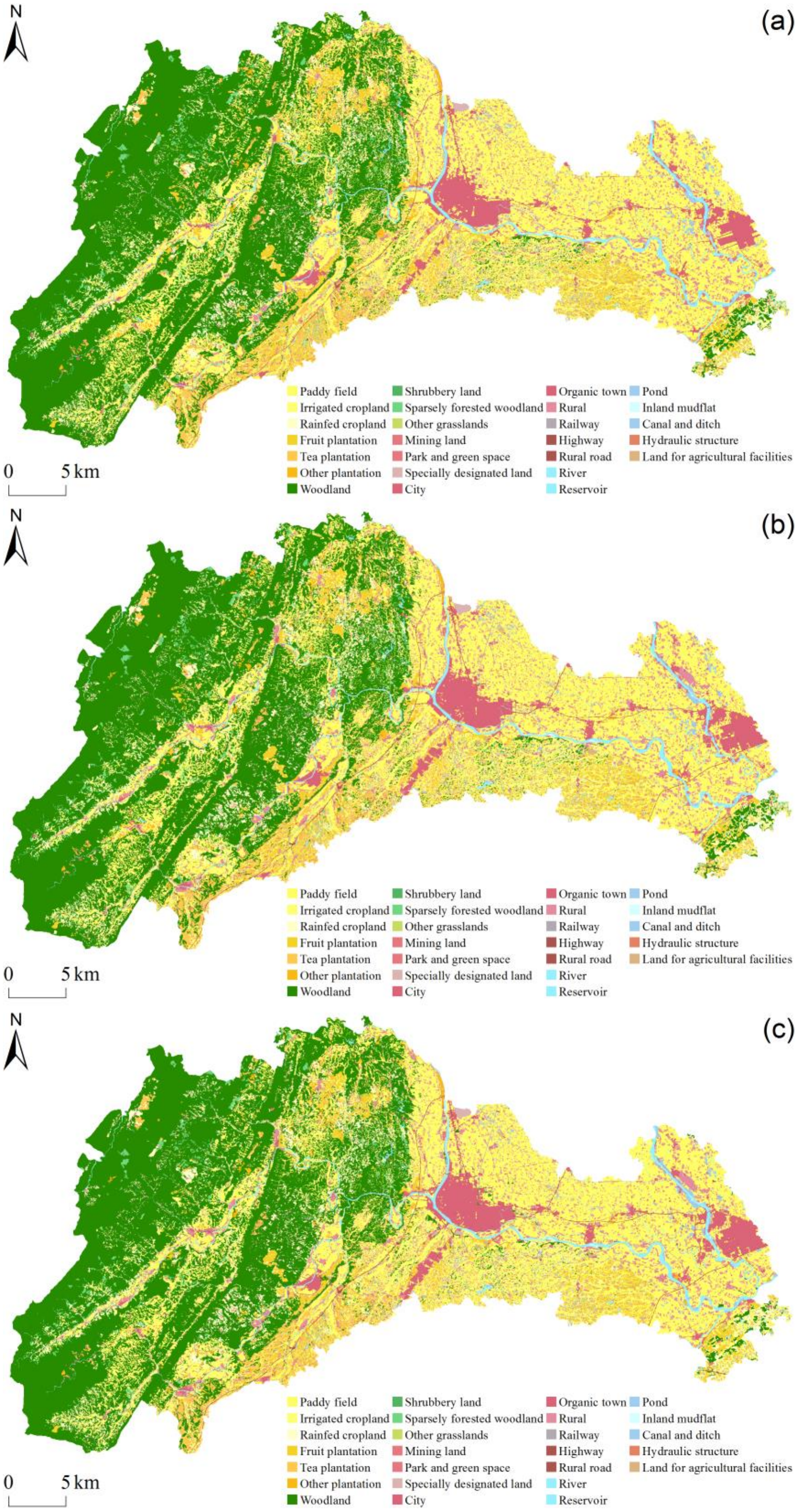

Figure S2. Spatial distribution of the first-level types of territorial space classification in Qionglai. (a, b, c) are spatial distribution map of the first-level types of territorial space classification in 2010, 2015 and 2020.

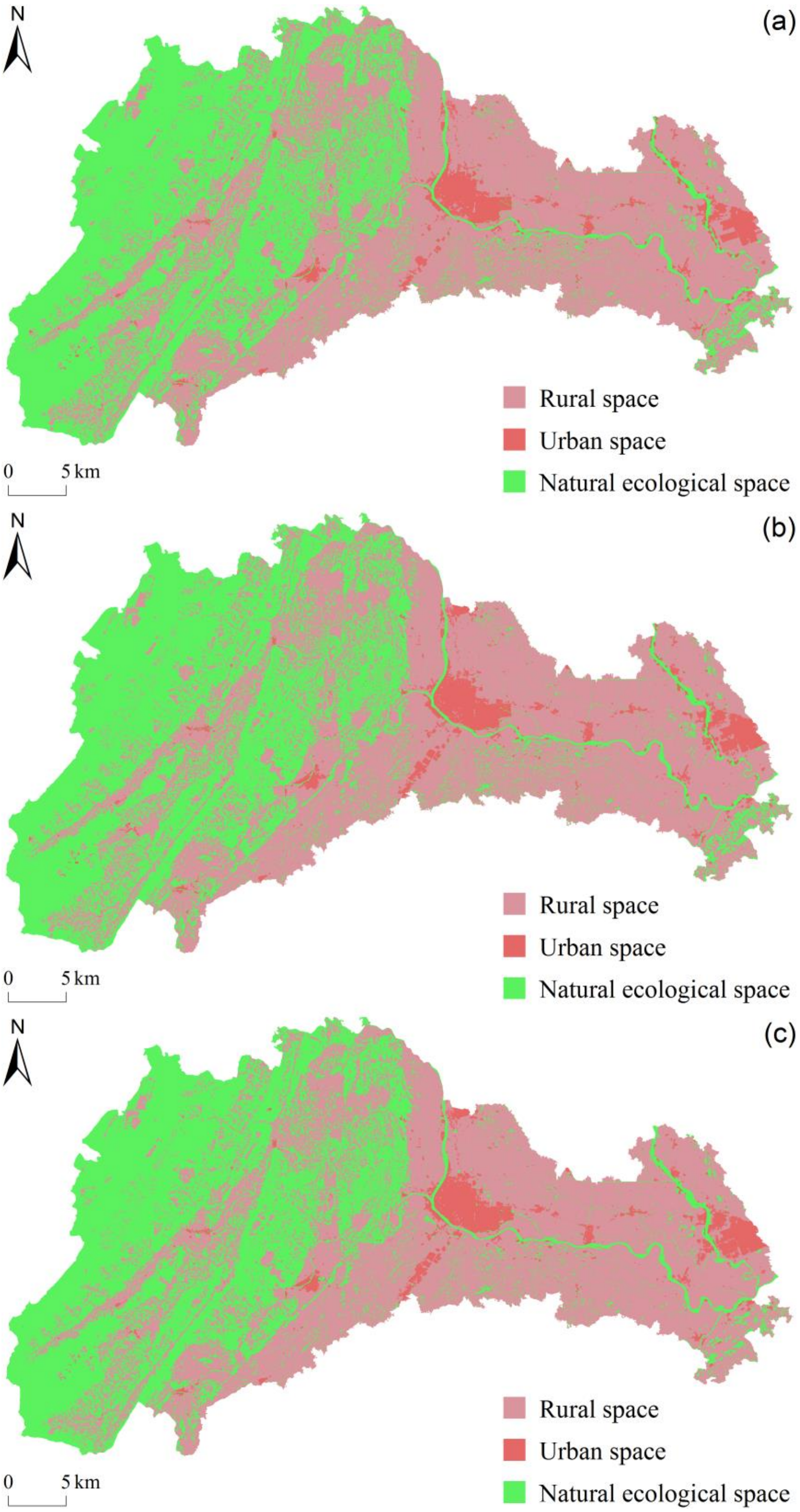

Figure S3. Spatial distribution of the second-level types of territorial space classification in Qionglai. (a, b, c) are spatial distribution map of the second-level types of territorial space classification in 2010, 2015 and 2020.

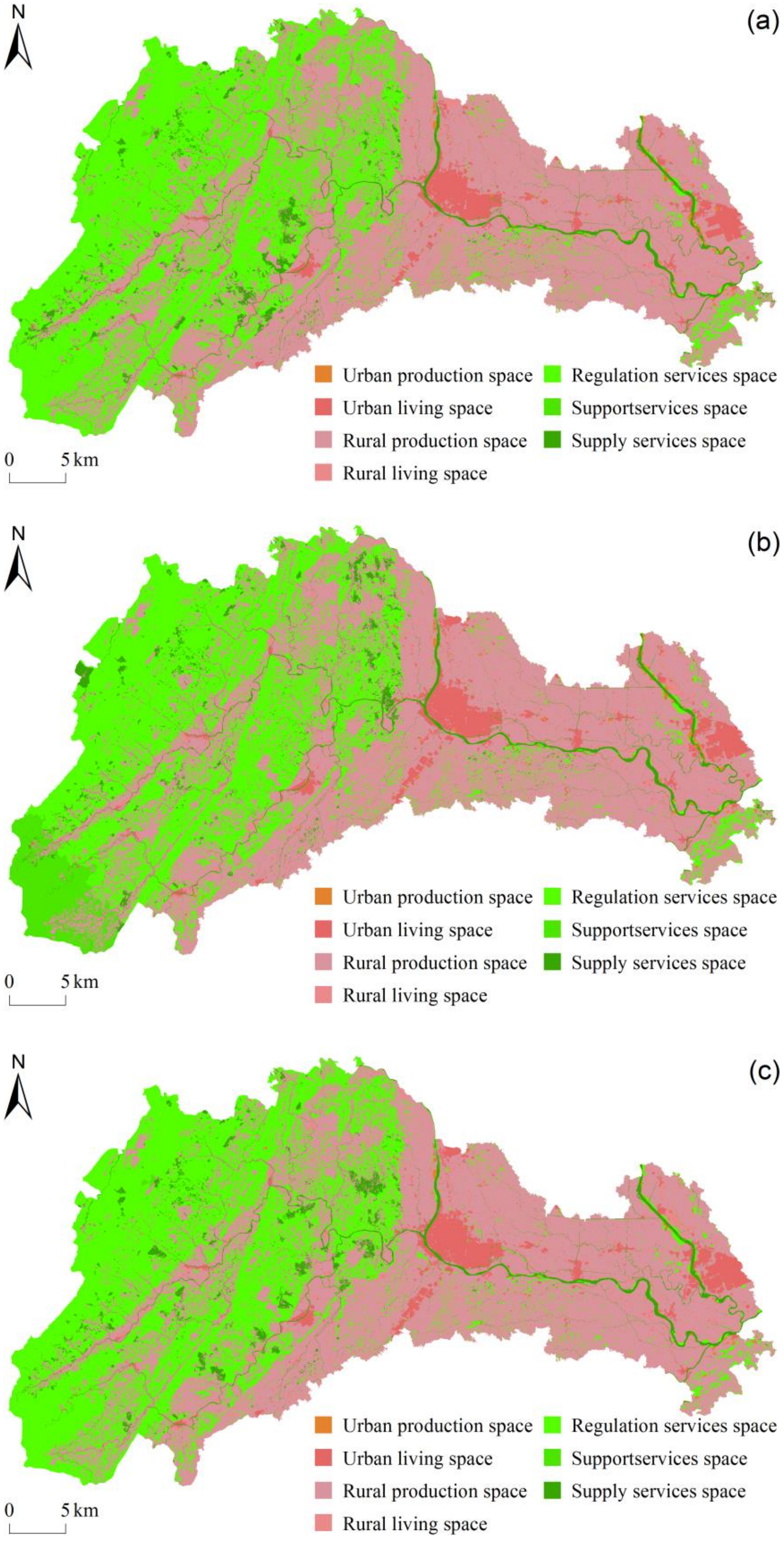

Table S1. Description of the third-level types of territorial space classification in Table 2

| Code | Name | Description |
|---|---|---|
| U11 | Industrial products supply | Areas mainly used in industrial production such as mining, manufacturing and so on, as well as providing material storage, transit transportation and so on. |
| U12 | Service industrial products supply | Areas mainly used in commercial and service industries such as wholesale and retail, accommodation and catering, business finance and so on. |
| U21 | Urban residential carrying | Areas that mainly provide living places for urban residents. |
| U22 | Urban living security | Areas that mainly provide urban residents with public service facilities such as travel, education, medical and health, as well as ecological services and employment guarantees. |
| R11 | Agricultural products supply | Areas mainly used for the production of primary agricultural products such as grain, oil, cotton, meat, eggs, milk, livestock, and aquatic products and so on. |
| R12 | Transportation services supply | Areas such as ground lines (railroads, highways, etc.) and stations (ports, wharves, etc.) used for transportation outside of towns and villages. |
| R21 | Rural residential carrying | Areas that mainly provide living places for rural residents. |
| R22 | Rural living security | Areas that mainly provide rural residents with public service facilities such as travel, education, medical and health, as well as ecological services and employment guarantees. |
| E11 | Raw materials production | Areas with the main major ecosystem service capabilities, such as converting solar energy into animal and plant products and biological energy and so on. |
| E12 | Water supply | Areas with the main major ecosystem service capabilities, such as freshwater filtration, retention, storage and supply and so on. |
| E21 | Gas regulation | Ecological areas that play a major role in regulating regional atmospheric chemical components (fixing $CO^2$ and releasing $O^2$). |
| E22 | Climate regulation | Ecological areas that play a major role in regulating of regional climate, such as increase precipitation and decrease temperature. |
| E23 | Environmental purification | Ecological areas that play a major role in regional environmental purification (e.g., purification of water and air, remove excess nutrients and compounds by biological decomposition, and retention of dust in vegetation, etc.). |
| E31 | Soil conservation | Areas with the main ecosystem service capabilities such as maintaining soil fertility (nutrient cycling and accumulation) and controlling soil erosion and landslides and so on. |
| E32 | Biodiversity maintenance | Areas with the main major ecosystem service capabilities, such as providing habitats for wild animals and plants and maintaining biodiversity and so on. |
| E41 | Aesthetic landscape | Areas with dominant landscape functions including potential recreational, cultural and artistic values, and so on. |

Table S2. The $q$-value hierarchical statistical information table in 2010

| Land use type | Function | $N_1$ | $N_2$ | $N_3$ | $N_4$ | $N_5$ | $\mu_1$ | $\mu_2$ | $\mu_3$ | $\mu_4$ | $\mu_5$ | $\sigma_1$ | $\sigma_2$ | $\sigma_3$ | $\sigma_4$ | $\sigma_5$ | $L$ | $N$ | $\bar{Y}$ | $\sigma^2$ | $q$-value | $F$ | $\lambda$ | $F_\alpha$ | $P$-value |
|---|---|---|---|---|---|---|---|---|---|---|---|---|---|---|---|---|---|---|---|---|---|---|---|---|---|
| Tea plantation | Agricultural products supply | 405 | 546 | 614 | 848 | 8625 | 1234.6 | 1237.7 | 1342.5 | 1348.7 | 3849.5 | 1725070.9 | 1958550.2 | 2409605.6 | 2086117.2 | 75523775.4 | 5 | 11038 | 3292.8 | 60576568.0 | $0.018^{***}$ | 51.412 | 0.005 | 2.376 | 0.000 |
| | Climate regulation | 115 | 214 | 222 | | | 712.6 | 924.1 | 865.8 | | | 335953.0 | 719916.1 | 572478.3 | | | 3 | 551 | 856.5 | 586504.7 | 0.010 | 2.894 | 0.010 | 3.027 | 0.057 |
| | Raw materials production | 19 | 30 | 8 | | | 803.0 | 1192.8 | 1312.3 | | | 303677.4 | 1447307.4 | 1429220.1 | | | 3 | 57 | 1079.6 | 1103412.7 | 0.036 | 1.012 | 0.431 | 3.816 | 0.443 |
| | Soil conservation | 13 | 14 | 52 | | | 574.0 | 500.5 | 819.4 | | | 149680.1 | 151069.5 | 353528.2 | | | 3 | 79 | 722.5 | 302651.6 | 0.061 | 2.481 | 0.076 | 3.234 | 0.098 |
| Canal and ditch | Climate regulation | 5 | 38 | 128 | | | 2165.7 | 3538.6 | 3754.8 | | | 5373493.1 | 18147325.0 | 36424753.4 | | | 3 | 171 | 3660.3 | 31530465.4 | 0.002 | 0.201 | 0.106 | 3.210 | 0.827 |
| | Biodiversity maintenance | 76 | 18 | 3 | | | 4423.9 | 1760.4 | 218.7 | | | 98801400.3 | 3637355.1 | 653.6 | | | 3 | 97 | 3799.6 | 79560003.3 | 0.019 | 0.887 | 0.006 | 3.103 | 0.416 |
| | Water supply | 63 | 61 | 35 | | | 5846.1 | 6633.3 | 15877.2 | | | 73420268.3 | 86434696.9 | 503591790.4 | | | 3 | 159 | 8356.2 | 189191959.2 | $0.085^{**}$ | 7.249 | 0.518 | 3.796 | 0.003 |
| Shrubbery land | Biodiversity maintenance | 16 | 88 | 53 | | | 2351.5 | 5640.4 | 6705.2 | | | 8428869.8 | 34555667.5 | 153589369.6 | | | 3 | 157 | 5664.7 | 73560916.2 | 0.020 | 1.586 | 0.049 | 2.395 | 0.216 |
| | Soil conservation | 6 | 2 | 6 | 38 | | 1130.2 | 1412.2 | 8638.2 | 2945.7 | | 1530862.0 | 27744.7 | 33727397.1 | 15644799.3 | | 4 | 52 | 3334.1 | 19560970.7 | $0.208^{*}$ | 4.189 | 2.515 | 3.866 | 0.077 |
| Fruit plantation | Climate regulation | 134 | 260 | 736 | 953 | | 826.2 | 902.8 | 834.7 | 1112.3 | | 583875.2 | 758464.1 | 719311.9 | 1221087.5 | | 4 | 2083 | 969.7 | 962685.9 | $0.018^{***}$ | 12.929 | 0.284 | 2.850 | 0.000 |
| | Raw materials production | 48 | 58 | 30 | | | 1044.1 | 1459.0 | 1670.7 | | | 1064994.5 | 2664468.7 | 3007908.8 | | | 3 | 136 | 1359.3 | 2236397.3 | 0.027 | 1.855 | 0.202 | 3.366 | 0.189 |
| | Agricultural products supply | 724 | 994 | 1149 | 1341 | 8955 | 1462.8 | 1734.5 | 1796.8 | 1955.7 | 5819.6 | 2329457.0 | 3594157.6 | 4084994.3 | 5266042.5 | 270558830.5 | 5 | 13163 | 4526.7 | 188924295.7 | $0.019^{***}$ | 63.288 | 0.001 | 2.373 | 0.000 |
| | Soil conservation | 8 | 11 | 99 | | | 816.4 | 561.6 | 1063.2 | | | 404232.7 | 96429.7 | 1500457.6 | | | 3 | 118 | 999.7 | 1318806.7 | 0.018 | 1.046 | 0.204 | 3.380 | 0.391 |
| Rainfed cropland | Agricultural products supply | 214 | 433 | 623 | 785 | 9970 | 859.6 | 1087.0 | 1378.6 | 1487.8 | 5269.8 | 649727.0 | 949694.8 | 2161671.6 | 2381358.9 | 98598828.2 | 5 | 12025 | 4592.2 | 84297288.3 | $0.027^{***}$ | 81.850 | 0.000 | 2.373 | 0.000 |
| | Climate regulation | 9 | 11 | 29 | | | 498.5 | 510.0 | 489.5 | | | 59369.9 | 93273.6 | 91247.5 | | | 3 | 49 | 495.7 | 85917.6 | 0.001 | 0.019 | 0.654 | 4.169 | 0.986 |
| | Gas regulation | 12 | 19 | 19 | | | 544.8 | 680.7 | 531.4 | | | 225824.3 | 173833.1 | 48241.5 | | | 3 | 50 | 591.3 | 143510.5 | 0.034 | 0.835 | 0.099 | 3.351 | 0.457 |
| River | Climate regulation | 39 | 146 | 241 | | | 32947.1 | 14715.3 | 16903.0 | | | 2176413104.7 | 1340764280.8 | 2207599317.3 | | | 3 | 426 | 17622.0 | 1932349172.4 | 0.013 | 2.737 | 0.315 | 3.473 | 0.095 |
| | Biodiversity maintenance | 195 | 149 | 14 | | | 63423.7 | 39823.2 | 3187.4 | | | 9487078290.9 | 3755334300.7 | 22267745.6 | | | 3 | 358 | 51245.5 | 6956789834.6 | $0.032^{**}$ | 5.944 | 0.039 | 3.080 | 0.003 |
| | Water supply | 39 | 41 | | | | 58079.5 | 27624.2 | | | | 4740421714.1 | 1212251206.0 | | | | 2 | 80 | 42471.2 | 3163970003.0 | $0.073^{**}$ | 6.164 | 0.157 | 4.570 | 0.023 |
| Pond | Climate regulation | 191 | 263 | 584 | 487 | | 347.9 | 525.1 | 508.3 | 561.8 | | 90632.5 | 350331.8 | 478187.3 | 435321.4 | | 4 | 1525 | 508.2 | 398095.8 | $0.011^{***}$ | 5.389 | 0.059 | 2.662 | 0.000 |
| | Biodiversity maintenance | 954 | 1442 | 729 | 57 | | 1692.5 | 2279.5 | 1452.3 | 395.5 | | 3609327.2 | 4436286.1 | 2279793.0 | 52912.7 | | 4 | 3182 | 1880.3 | 3780040.8 | $0.043^{***}$ | 48.125 | 0.007 | 2.614 | 0.000 |
| | Environmental purification | 9 | 120 | 344 | | | 4762.9 | 6277.9 | 15908.0 | | | 6664890.8 | 15475719.5 | 211173901.4 | | | 3 | 473 | 13252.8 | 176475189.6 | $0.107^{***}$ | 28.088 | 0.073 | 3.124 | 0.000 |
| | Water supply | 31 | 90 | 43 | | | 1262.1 | 1910.3 | 1932.4 | | | 1496232.7 | 4731825.4 | 2820850.0 | | | 3 | 164 | 1793.5 | 3685081.0 | 0.018 | 1.466 | 0.069 | 3.156 | 0.245 |
| Inland mudflat | Biodiversity maintenance | 2 | 16 | 11 | 6 | 12 | 1909.7 | 18862.5 | 33575.3 | 6652.3 | 2654.3 | 2739338.2 | 542545483.0 | 1049959126.0 | 27088446.0 | 8332610.9 | 5 | 47 | 15887.5 | 576281203.2 | $0.243^{**}$ | 3.374 | 0.962 | 3.184 | 0.040 |
| | Aesthetic landscape | 3 | 3 | 5 | | | 3635.9 | 4457.1 | 48662.3 | | | 4152886.2 | 3358888.0 | 2795462692.7 | | | 3 | 11 | 24326.4 | 1766334640.4 | 0.279 | 1.551 | 0.583 | 5.701 | 0.346 |
| Other grasslands | Biodiversity maintenance | 2 | 18 | | | | 148.0 | 2567.4 | | | | 7.0 | 4616009.8 | | | | 2 | 20 | 2325.4 | 4681217.3 | 0.113 | 2.283 | 0.096 | 4.833 | 0.166 |
| | Soil conservation | 35 | 18 | | | | 5045.6 | 1629.2 | | | | 37698284.0 | 1322754.4 | | | | 2 | 53 | 3885.3 | 27961971.9 | $0.094^{**}$ | 5.267 | 0.093 | 4.402 | 0.032 |
| Sparsely forested woodland | Raw materials production | 212 | 236 | 240 | 217 | 115 | 13652.7 | 8664.3 | 8197.6 | 8267.4 | 9638.8 | 968725513.0 | 301668285.0 | 403894640.6 | 259954578.8 | 335659371.4 | 5 | 1020 | 9616.7 | 463779573.0 | $0.010^{**}$ | 2.462 | 0.067 | 2.421 | 0.047 |
| | Climate regulation | 393 | 236 | | | | 8027.4 | 3356.9 | | | | 181296248.4 | 48282219.4 | | | | 2 | 629 | 6275.0 | 136503054.7 | $0.037^{***}$ | 24.402 | 0.038 | 4.000 | 0.000 |
| | Soil conservation | 1 | 217 | | | | 31941.9 | 4230.5 | | | | 0.0 | 33653003.8 | | | | 2 | 218 | 4357.7 | 37005050.3 | 0.095 | 22.609 | 26.954 | 48.068 | 0.663 |
| Other orchards | Raw materials production | 13 | 11 | 4 | | | 988.1 | 960.0 | 860.2 | | | 580427.8 | 455719.3 | 171268.4 | | | 3 | 28 | 958.8 | 474771.1 | $0.004^{***}$ | 0.047 | 0.163 | 3.657 | 0.957 |
| | Agricultural products supply | 288 | 367 | 503 | 4765 | | 1306.2 | 1240.0 | 1405.6 | 3244.9 | | 1874367.5 | 2172436.8 | 2589249.2 | 54785122.3 | | 4 | 5923 | 2870.2 | 45098462.6 | 0.013 | 25.646 | 0.009 | 2.614 | 0.000 |
| | Climate regulation | 55 | 154 | 294 | | | 794.2 | 805.4 | 995.2 | | | 299202.2 | 512853.9 | 987014.5 | | | 3 | 503 | 915.1 | 775665.1 | 0.012 | 2.944 | 0.144 | 3.227 | 0.065 |
| | Soil conservation | 4 | 8 | 59 | | | 720.7 | 687.7 | 970.3 | | | 366244.4 | 329840.3 | 973778.0 | | | 3 | 71 | 924.4 | 877398.5 | 0.012 | 0.408 | 0.318 | 3.611 | 0.706 |
| Irrigated cropland | Raw materials production | 54 | 73 | 41 | | | 569.3 | 542.9 | 614.0 | | | 164432.2 | 164250.5 | 203890.1 | | | 3 | 168 | 568.8 | 174773.5 | 0.005 | 0.375 | 0.158 | 3.288 | 0.708 |
| | Agricultural products supply | 2068 | 2711 | 3240 | 3755 | 18226 | 821.0 | 1044.4 | 1240.4 | 1472.7 | 3719.0 | 565149.5 | 1133300.1 | 1867741.3 | 3102822.0 | 45083291.3 | 5 | 30000 | 2728.7 | 29660832.9 | $0.052^{***}$ | 410.596 | 0.011 | 2.379 | 0.000 |
| | Climate regulation | 137 | 643 | 1537 | 532 | | 536.3 | 579.2 | 508.8 | 451.2 | | 143636.9 | 248845.3 | 151716.1 | 133031.0 | | 4 | 2849 | 515.3 | 171494.5 | $0.010^{***}$ | 9.690 | 0.940 | 3.360 | 0.000 |
| | Soil conservation | 48 | 34 | 176 | | | 555.3 | 554.0 | 689.4 | | | 267740.9 | 274148.7 | 340052.2 | | | 3 | 258 | 646.6 | 321842.2 | 0.012 | 1.575 | 0.219 | 3.354 | 0.244 |
| Paddy field | Agricultural products supply | 957 | 1338 | 1763 | 2265 | 23677 | 1390.6 | 1706.3 | 1967.9 | 2186.2 | 4516.3 | 1450787.4 | 2066111.4 | 3289134.9 | 4103823.9 | 35809254.6 | 5 | 30000 | 3965.6 | 30055522.6 | $0.038^{***}$ | 298.904 | 0.047 | 2.400 | 0.000 |
| | Climate regulation | 237 | 546 | 1008 | 564 | | 863.1 | 917.8 | 1003.2 | 893.2 | | 439821.7 | 1246046.0 | 560244.3 | 578055.0 | | 4 | 2355 | 942.9 | 714325.5 | $0.004^{**}$ | 3.232 | 0.166 | 2.751 | 0.027 |
| Woodland | Raw materials production | 55 | 83 | 155 | 183 | 178 | 6524.8 | 9584.2 | 6760.6 | 11593.7 | 37420.1 | 175554944.6 | 2214025977.7 | 199826276.7 | 846739898.9 | 3969146420.2 | 5 | 654 | 16796.1 | 1823011092.4 | $0.089^{***}$ | 15.898 | 0.297 | 2.559 | 0.000 |
| | Climate regulation | 1789 | 3311 | 2920 | 2567 | 2967 | 61454.1 | 64945.9 | 33779.0 | 18137.2 | 12741.3 | 80467257654.7 | 57434091405.1 | 11152431053.0 | 2205619560.2 | 1298775058.8 | 5 | 13554 | 37477.8 | 28223610586.8 | $0.017^{***}$ | 57.104 | 0.091 | 2.426 | 0.000 |
| | Biodiversity maintenance | 92 | 4 | 237 | 275 | 2 | 1381.1 | 1271.2 | 7023.4 | 7306.5 | 1517.1 | 4184446.8 | 419902.7 | 118777236.9 | 238187952.1 | 37314.1 | 5 | 610 | 6244.3 | 158708622.8 | $0.029^{***}$ | 4.461 | 0.053 | 2.418 | 0.000 |
| | Soil conservation | 24 | 20 | 603 | | | 12025.5 | 8908.4 | 3530.0 | | | 466990766.3 | 222260292.1 | 32470273.9 | | | 3 | 647 | 4011.4 | 57794954.4 | $0.058^{***}$ | 19.748 | 3.171 | 6.601 | 0.000 |

Note: $N_h$ is the number of patches on land use map in $h = 1,2,\cdots,L$ stratum of a certain function in one type of land use; $\mu_h$ is the $h$ stratum mean of the patch areas of a certain function in one type of land use; $\sigma_h$ is the $h$ stratum variance of the patch areas of a certain function in one type of land use; $L, N$ are the stratum number, total number of patches of a certain function in one type of land use, respectively; $\bar{Y}, \sigma^2$ are the population mean, variance of the patch areas of a certain function in one type of land use, respectively; $F, P$-value are the testing statistics of the noncentral $F$-distribution; $\lambda$ is the value of the noncentral parameter; $F_\alpha$ is the critical value at significant level $\alpha$. Superscript *, ** and *** of $q$-value denote statistical significance at level of $\alpha = 0.1, 0.05$ and $0.001$, respectively.

Table S3. The $q$-value hierarchical statistical information table in 2015

| Land use type | Function | $N_1$ | $N_2$ | $N_3$ | $N_4$ | $N_5$ | $\mu_1$ | $\mu_2$ | $\mu_3$ | $\mu_4$ | $\mu_5$ | $\sigma_1$ | $\sigma_2$ | $\sigma_3$ | $\sigma_4$ | $\sigma_5$ | $L$ | $N$ | $\bar{Y}$ | $\sigma^2$ | $q$-value | $F$ | $\lambda$ | $F_\alpha$ | $P$-value |
|---|---|---|---|---|---|---|---|---|---|---|---|---|---|---|---|---|---|---|---|---|---|---|---|---|---|
| Tea plantation | Agricultural products supply | 406 | 520 | 582 | 771 | 7667 | 1117.0 | 1255.0 | 1370.3 | 1381.3 | 3962.5 | 1317671.3 | 1925625.7 | 2398044.3 | 2210608.4 | 81773866.5 | 5 | 9946 | 3353.0 | 64754503.6 | $0.019^{***}$ | 48.996 | 0.002 | 2.374 | 0.000 |
| | Climate regulation | 110 | 238 | 134 | | | 692.4 | 962.2 | 771.8 | | | 343935.0 | 779337.8 | 431977.3 | | | 3 | 482 | 847.7 | 596976.5 | $0.023^{**}$ | 5.572 | 0.003 | 3.020 | 0.004 |
| | Raw materials production | 43 | 33 | 32 | | | 769.9 | 882.1 | 816.2 | | | 497593.9 | 523899.4 | 704742.3 | | | 3 | 108 | 817.9 | 569184.7 | 0.004 | 0.201 | 0.048 | 3.156 | 0.822 |
| | Soil conservation | 5 | 6 | 20 | | | 557.5 | 1266.2 | 723.0 | | | 263820.5 | 1967108.9 | 283252.5 | | | 3 | 31 | 801.5 | 661402.1 | 0.084 | 1.279 | 0.881 | 4.678 | 0.429 |
| Canal and ditch | Climate regulation | 3 | 46 | 4 | | | 7638.1 | 1920.1 | 2396.2 | | | 6854071.4 | 9809032.1 | 3555364.6 | | | 3 | 53 | 2279.7 | 10908301.3 | 0.159 | 4.740 | 4.546 | 8.425 | 0.237 |
| | Biodiversity maintenance | 17 | 26 | 1 | | | 2299.1 | 2074.7 | 194.1 | | | 5133850.9 | 4813142.2 | 0.0 | | | 3 | 44 | 2118.7 | 4925558.1 | 0.020 | 0.416 | 0.062 | 3.325 | 0.671 |
| | Water supply | 140 | 140 | 80 | | | 3279.6 | 5581.3 | 10534.8 | | | 26523258.7 | 51109535.1 | 309492672.8 | | | 3 | 360 | 5787.0 | 106437487.5 | $0.070^{***}$ | 13.475 | 0.402 | 3.598 | 0.000 |
| Shrubbery land | Biodiversity maintenance | 9 | 14 | 27 | | | 1496.9 | 8291.0 | 5230.4 | | | 1097351.0 | 59683383.5 | 29714509.2 | | | 3 | 50 | 5415.3 | 38052382.4 | $0.134^{*}$ | 3.635 | 0.519 | 3.030 | 0.063 |
| | Soil conservation | 23 | 26 | 27 | 35 | 52 | 9026.2 | 5760.1 | 2908.9 | 4410.9 | 3878.4 | 299598091.1 | 37617366.1 | 15328421.4 | 28430544.5 | 18881872.0 | 5 | 163 | 4858.7 | 66501958.6 | 0.054 | 2.234 | 0.514 | 2.730 | 0.099 |
| Fruit plantation | Climate regulation | 470 | 296 | 241 | 247 | | 757.9 | 1119.5 | 1078.9 | 886.6 | | 657523.2 | 1356696.0 | 861124.7 | 678272.9 | | 4 | 1254 | 930.3 | 889975.5 | $0.027^{***}$ | 11.647 | 0.297 | 2.864 | 0.000 |
| | Raw materials production | 189 | 221 | 88 | | | 892.5 | 1052.0 | 1557.7 | | | 633977.4 | 1030940.2 | 2711984.8 | | | 3 | 498 | 1080.8 | 1231355.3 | $0.044^{***}$ | 11.356 | 0.568 | 3.810 | 0.000 |
| | Agricultural products supply | 647 | 897 | 1083 | 1302 | 8012 | 1336.0 | 1693.7 | 1851.7 | 1981.1 | 5859.2 | 1934599.5 | 3124072.9 | 4327780.3 | 5256453.6 | 282199447.2 | 5 | 11941 | 4514.9 | 194352565.6 | $0.019^{***}$ | 57.931 | 0.001 | 2.374 | 0.000 |
| | Soil conservation | 6 | 10 | 53 | | | 607.4 | 2315.9 | 992.5 | | | 64474.5 | 7419588.2 | 876678.1 | | | 3 | 69 | 1150.8 | 1995966.4 | 0.121 | 4.546 | 1.498 | 5.135 | 0.071 |
| Rainfed cropland | Agricultural products supply | 488 | 757 | 1030 | 1337 | 12858 | 776.7 | 994.5 | 1255.3 | 1359.8 | 4566.5 | 514549.0 | 1003772.3 | 1793869.5 | 2306166.6 | 81368508.1 | 5 | 16470 | 3822.7 | 65863679.8 | $0.030^{***}$ | 127.522 | 0.000 | 2.373 | 0.000 |
| | Climate regulation | 63 | 33 | 17 | | | 479.6 | 398.4 | 463.6 | | | 82705.6 | 58024.6 | 65884.0 | | | 3 | 113 | 453.4 | 74249.7 | 0.017 | 0.967 | 0.489 | 3.788 | 0.467 |
| | Gas regulation | 80 | 77 | 55 | | | 457.3 | 548.4 | 620.4 | | | 159884.7 | 166609.6 | 170883.9 | | | 3 | 212 | 532.7 | 169412.3 | 0.025 | 2.677 | 0.205 | 3.342 | 0.090 |
| River | Climate regulation | 138 | 140 | 38 | | | 9181.8 | 5488.7 | 19405.2 | | | 321038761.4 | 144865046.5 | 1054035512.5 | | | 3 | 316 | 8775.0 | 349578067.4 | $0.053^{***}$ | 8.718 | 0.630 | 3.905 | 0.000 |
| | Biodiversity maintenance | 132 | 82 | 1 | | | 12743.2 | 32826.7 | 226.1 | | | 1090094760.7 | 2182745064.6 | 0.0 | | | 3 | 215 | 20344.7 | 1598536067.9 | $0.061^{**}$ | 6.831 | 0.202 | 3.338 | 0.002 |
| | Water supply | 60 | 179 | 109 | | | 19183.2 | 90338.4 | 43267.3 | | | 775340014.7 | 9441576979.8 | 5366608774.6 | | | 3 | 348 | 63326.7 | 7508350707.4 | $0.112^{***}$ | 21.651 | 0.133 | 3.219 | 0.000 |
| Pond | Climate regulation | 156 | 475 | 408 | 321 | | 395.3 | 513.3 | 476.4 | 380.3 | | 106587.5 | 1034322.8 | 210254.8 | 88453.0 | | 4 | 1360 | 457.3 | 460475.5 | $0.007^{**}$ | 3.007 | 0.026 | 2.634 | 0.030 |
| | Biodiversity maintenance | 67 | 1907 | 740 | 41 | | 493.6 | 2616.8 | 1657.9 | 378.6 | | 212777.2 | 13317392.3 | 6731595.4 | 46256.6 | | 4 | 2755 | 2274.3 | 11346077.7 | $0.028^{***}$ | 26.087 | 0.000 | 2.608 | 0.000 |
| | Environmental purification | 29 | 90 | | | | 15872.8 | 24361.3 | | | | 173667799.5 | 448786217.8 | | | | 2 | 119 | 22292.7 | 395021092.2 | $0.034^{**}$ | 4.070 | 0.008 | 3.954 | 0.047 |
| | Water supply | 84 | 395 | 373 | 225 | | 1834.8 | 2984.9 | 4472.2 | 2770.3 | | 8335051.0 | 9670216.5 | 36450809.6 | 8903561.1 | | 4 | 1077 | 3365.5 | 19414977.9 | $0.038^{***}$ | 14.055 | 0.066 | 2.671 | 0.000 |
| Inland mudflat | Biodiversity maintenance | 2 | 24 | 22 | | | 1909.7 | 16103.2 | 5462.4 | | | 2739338.2 | 447467014.4 | 62806457.4 | | | 3 | 48 | 10634.8 | 283020142.0 | $0.107^{*}$ | 2.706 | 0.188 | 2.651 | 0.095 |
| | Aesthetic landscape | 2 | 8 | | | | 9509.0 | 60602.1 | | | | 12860075.5 | 2035833438.2 | | | | 2 | 10 | 50383.5 | 2048919732.2 | 0.204 | 2.048 | 0.169 | 6.198 | 0.220 |
| Other grasslands | Biodiversity maintenance | 5 | 43 | 3 | | | 659.6 | 4476.7 | 1179.3 | | | 288580.8 | 31369687.6 | 109138.9 | | | 3 | 51 | 3908.5 | 28228848.9 | 0.062 | 1.582 | 0.024 | 3.229 | 0.220 |
| | Soil conservation | 5 | 5 | 5 | 6 | | 2207.4 | 5503.6 | 1427.2 | 1060.1 | | 4030942.8 | 9241587.9 | 287901.4 | 572046.0 | | 4 | 21 | 2478.6 | 6426407.3 | $0.472^{**}$ | 5.069 | 2.033 | 3.974 | 0.049 |
| Sparsely forested woodland | Raw materials production | 316 | 555 | 284 | | | 7883.1 | 5728.3 | 5948.4 | | | 257619046.8 | 102810846.7 | 183621629.5 | | | 3 | 1155 | 6371.9 | 165903586.0 | $0.005^{*}$ | 3.029 | 0.050 | 2.365 | 0.052 |
| | Climate regulation | 340 | 360 | | | | 14417.5 | 5057.9 | | | | 968787160.8 | 66086329.8 | | | | 2 | 700 | 9604.0 | 526423349.4 | $0.042^{***}$ | 30.273 | 0.088 | 4.190 | 0.000 |
| | Soil conservation | 1 | 2 | 87 | | | 6901.2 | 8977.6 | 6652.9 | | | 0.0 | 6466522.1 | 81404413.0 | | | 3 | 90 | 6707.4 | 78952450.3 | 0.001 | 0.065 | 1.246 | 4.780 | 0.965 |
| Other orchards | Raw materials production | 29 | 24 | 17 | | | 949.7 | 749.8 | 1089.4 | | | 575168.2 | 518114.9 | 728011.7 | | | 3 | 70 | 915.1 | 609966.6 | 0.028 | 0.974 | 0.216 | 3.464 | 0.420 |
| | Agricultural products supply | 226 | 346 | 499 | 3933 | | 1296.4 | 1251.8 | 1413.8 | 3475.2 | | 2216796.8 | 2242040.3 | 2776581.5 | 63569672.5 | | 4 | 5004 | 3017.5 | 51266457.1 | $0.015^{***}$ | 25.431 | 0.005 | 2.611 | 0.000 |
| | Climate regulation | 151 | 152 | 51 | | | 842.5 | 963.3 | 881.5 | | | 808868.6 | 726997.6 | 341744.5 | | | 3 | 354 | 900.0 | 709594.0 | 0.004 | 0.789 | 0.164 | 3.264 | 0.484 |
| | Soil conservation | 6 | 3 | 29 | | | 1234.2 | 766.2 | 913.5 | | | 782989.2 | 390812.3 | 618053.2 | | | 3 | 38 | 952.5 | 642590.0 | 0.026 | 0.459 | 1.064 | 4.816 | 0.757 |
| Irrigated cropland | Raw materials production | 479 | 523 | 435 | 219 | | 487.0 | 539.5 | 540.6 | 546.4 | | 136392.3 | 191789.7 | 222542.6 | 227678.5 | | 4 | 1656 | 525.5 | 189198.6 | 0.003 | 1.776 | 0.188 | 2.771 | 0.171 |
| | Agricultural products supply | 1941 | 2666 | 3336 | 3999 | 18058 | 799.4 | 1025.0 | 1194.3 | 1413.6 | 3727.1 | 510493.7 | 1125530.5 | 1958674.4 | 2823196.4 | 45163016.6 | 5 | 30000 | 2707.5 | 29503066.7 | $0.054^{***}$ | 427.362 | 0.013 | 2.380 | 0.000 |
| | Climate regulation | 74 | 994 | 401 | 227 | | 432.8 | 497.7 | 486.4 | 531.7 | | 129689.4 | 155155.9 | 139658.6 | 144906.4 | | 4 | 1696 | 496.8 | 149376.4 | 0.002 | 1.391 | 0.936 | 3.361 | 0.369 |
| | Soil conservation | 36 | 33 | 108 | | | 543.6 | 486.6 | 663.1 | | | 240915.2 | 100475.1 | 349783.0 | | | 3 | 177 | 605.9 | 286598.7 | 0.019 | 1.683 | 0.087 | 3.178 | 0.202 |
| Paddy field | Agricultural products supply | 935 | 1312 | 1871 | 2438 | 23444 | 1248.5 | 1599.3 | 1851.7 | 2089.7 | 4521.6 | 1165863.1 | 1776713.9 | 2727370.2 | 3643463.7 | 35436213.3 | 5 | 30000 | 3927.6 | 29552207.2 | $0.043^{***}$ | 339.433 | 0.030 | 2.390 | 0.000 |
| | Climate regulation | 169 | 1418 | 622 | 307 | | 789.6 | 838.9 | 852.5 | 873.8 | | 331068.7 | 763421.8 | 515012.7 | 528837.7 | | 4 | 2516 | 843.2 | 644684.6 | 0.001 | 0.440 | 0.578 | 3.086 | 0.778 |
| Woodland | Raw materials production | 98 | 137 | 209 | 363 | | 3224.5 | 17701.1 | 4718.4 | 19887.2 | | 18136979.2 | 26237660162.4 | 81169614.8 | 1822023374.5 | | 4 | 807 | 13564.1 | 5351157594.2 | $0.010^{**}$ | 2.736 | 0.030 | 2.642 | 0.044 |
| | Climate regulation | 3046 | 3947 | 4189 | 2948 | 1453 | 24677.0 | 38775.0 | 30267.8 | 22622.9 | 23366.4 | 13928891591.6 | 34209698208.4 | 9737900909.5 | 3399586375.4 | 4546849702.8 | 5 | 15583 | 29240.0 | 15111314058.9 | $0.003^{***}$ | 10.047 | 0.007 | 2.377 | 0.000 |
| | Biodiversity maintenance | 28 | 268 | 284 | | | 1024.8 | 30203.5 | 161036.7 | | | 1289826.0 | 4825906738.2 | 479603941049.9 | | | 3 | 580 | 92858.0 | 241567617331.9 | $0.019^{**}$ | 5.473 | 0.037 | 3.067 | 0.005 |
| | Soil conservation | 15 | 23 | 762 | | | 11164.2 | 12220.0 | 4991.7 | | | 641462993.4 | 2226552226.0 | 390914727.6 | | | 3 | 800 | 5315.2 | 450498934.4 | 0.005 | 1.877 | 0.504 | 3.718 | 0.225 |

Note: "$N_1, N_2, \cdots, N_5$, $\mu_1, \mu_2, \cdots, \mu_5$, $\sigma_1, \sigma_2, \cdots, \sigma_5$, $L$, $N$, $\bar{Y}$, $\sigma^2$, $q$-value, $F$, $\lambda$, $F_\alpha$, $P$-value" have the meanings stated in Table S2.

Table S4. The $q$-value hierarchical statistical information table in 2020

| Land use type | Function | $N_1$ | $N_2$ | $N_3$ | $N_4$ | $N_5$ | $\mu_1$ | $\mu_2$ | $\mu_3$ | $\mu_4$ | $\mu_5$ | $\sigma_1$ | $\sigma_2$ | $\sigma_3$ | $\sigma_4$ | $\sigma_5$ | $L$ | $N$ | $\bar{Y}$ | $\sigma^2$ | $q$-value | $F$ | $\lambda$ | $F_\alpha$ | $P$-value |
|---|---|---|---|---|---|---|---|---|---|---|---|---|---|---|---|---|---|---|---|---|---|---|---|---|---|
| Tea plantation | Agricultural products supply | 325 | 454 | 477 | 654 | 6590 | 1110.9 | 1301.4 | 1364.3 | 1446.6 | 4000.9 | 1455696.5 | 2108558.7 | 2135042.8 | 2431643.7 | 78789585.8 | 5 | 8500 | 3401.8 | 62802003.9 | 0.020$^{***}$ | 42.838 | 0.003 | 2.375 | 0.000 |
| | Climate regulation | 62 | 133 | 174 | | | 751.7 | 841.0 | 907.6 | | | 450190.9 | 455240.6 | 580430.0 | | | 3 | 369 | 857.4 | 516589.6 | 0.006 | 1.128 | 0.064 | 3.117 | 0.336 |
| | Raw materials production | 99 | 38 | 8 | | | 901.2 | 1211.4 | 685.7 | | | 556207.0 | 2134088.0 | 110418.8 | | | 3 | 145 | 970.6 | 968083.7 | 0.024 | 1.725 | 0.435 | 3.691 | 0.247 |
| | Soil conservation | 7 | 9 | 26 | | | 468.4 | 998.5 | 800.6 | | | 42852.8 | 1741441.0 | 253649.2 | | | 3 | 42 | 787.6 | 563946.2 | 0.047 | 0.966 | 0.373 | 3.816 | 0.452 |
| Canal and ditch | Climate regulation | 21 | 67 | 52 | | | 2164.6 | 2525.4 | 3252.1 | | | 14315181.3 | 9253618.4 | 19493743.0 | | | 3 | 140 | 2741.2 | 13985427.7 | 0.012 | 0.838 | 0.056 | 3.147 | 0.444 |
| | Biodiversity maintenance | 43 | 26 | 6 | | | 3775.5 | 4807.0 | 2131.7 | | | 27424735.5 | 67845936.4 | 3573914.2 | | | 3 | 75 | 4001.6 | 40063257.3 | 0.013 | 0.486 | 0.058 | 3.214 | 0.625 |
| | Water supply | 90 | 119 | 23 | | | 4047.0 | 7489.7 | 15458.1 | | | 42331719.4 | 131170707.9 | 428126293.5 | | | 3 | 232 | 6944.1 | 136741866.5 | 0.077$^{***}$ | 9.617 | 1.088 | 4.486 | 0.000 |
| Shrubbery land | Biodiversity maintenance | 4 | 27 | 9 | | | 1706.9 | 5778.4 | 5226.9 | | | 948935.0 | 35045829.8 | 42971777.8 | | | 3 | 40 | 5247.2 | 34863428.5 | 0.041 | 0.799 | 0.095 | 3.404 | 0.473 |
| | Soil conservation | 23 | 44 | 31 | 32 | 28 | 6382.9 | 4389.7 | 5461.7 | 4041.1 | 1856.3 | 70137277.7 | 18932423.7 | 28003382.2 | 23875548.9 | 2140294.8 | 5 | 158 | 4370.6 | 28156778.3 | 0.070$^{**}$ | 2.870 | 0.502 | 2.726 | 0.041 |
| Fruit plantation | Climate regulation | 127 | 236 | 512 | 801 | | 811.8 | 950.2 | 787.1 | 1107.5 | | 570752.6 | 826966.7 | 709794.2 | 1155271.0 | | 4 | 1676 | 1974.7 | 949836.5 | 0.022$^{***}$ | 12.708 | 0.250 | 2.822 | 0.000 |
| | Raw materials production | 103 | 39 | 28 | | | 1152.6 | 1137.0 | 979.1 | | | 1446068.8 | 1089499.3 | 696089.6 | | | 3 | 170 | 1120.5 | 1244719.2 | 0.003 | 0.268 | 0.159 | 3.287 | 0.781 |
| | Agricultural products supply | 658 | 927 | 992 | 1188 | 7723 | 1461.9 | 1830.7 | 1798.1 | 2000.0 | 6026.4 | 2289001.1 | 3742653.5 | 4097122.9 | 5070569.3 | 304100406.5 | 5 | 11488 | 4644.9 | 209673534.2 | 0.019$^{***}$ | 54.774 | 0.001 | 2.374 | 0.000 |
| | Soil conservation | 9 | 15 | 61 | | | 1386.4 | 1452.5 | 1196.5 | | | 926823.8 | 3360384.4 | 1892618.3 | | | 3 | 85 | 1261.7 | 2060499.3 | 0.005 | 0.223 | 0.562 | 3.923 | 0.845 |
| Rainfed cropland | Agricultural products supply | 779 | 1140 | 1457 | 1916 | 13498 | 793.4 | 940.7 | 1177.5 | 1336.0 | 4345.0 | 508376.6 | 1016857.8 | 1577760.5 | 2304314.1 | 78978101.0 | 5 | 18790 | 3438.8 | 59280908.9 | 0.036$^{***}$ | 172.988 | 0.004 | 2.375 | 0.000 |
| | Climate regulation | 16 | 177 | 192 | | | 461.2 | 460.1 | 447.8 | | | 50255.5 | 102471.7 | 107118.6 | | | 3 | 385 | 454.0 | 102657.9 | 0.000 | 0.072 | 1.006 | 4.364 | 0.957 |
| | Gas regulation | 110 | 89 | 59 | | | 430.7 | 473.7 | 616.0 | | | 85964.2 | 114951.4 | 223362.1 | | | 3 | 258 | 487.9 | 132598.9 | 0.039$^{**}$ | 5.220 | 0.452 | 3.678 | 0.014 |
| River | Climate regulation | 37 | 173 | 146 | | | 12383.1 | 6573.4 | 14237.0 | | | 516542355.6 | 117798369.7 | 1582239623.2 | | | 3 | 356 | 10320.2 | 773382349.8 | 0.018 | 3.149 | 0.112 | 3.187 | 0.052 |
| | Biodiversity maintenance | 170 | 91 | 16 | | | 45903.7 | 27479.8 | 3683.4 | | | 6553144648.5 | 1677123540.2 | 23507747.8 | | | 3 | 277 | 37412.3 | 4716484650.3 | 0.030$^{**}$ | 4.264 | 0.023 | 3.064 | 0.016 |
| | Water supply | 15 | 56 | 148 | 81 | 42 | 10828.7 | 22134.5 | 55663.5 | 53720.6 | 39390.6 | 737889632.3 | 1125810404.4 | 6173483095.3 | 5604374509.2 | 3397947563.2 | 5 | 342 | 45748.3 | 4840263833.4 | 0.043$^{**}$ | 3.771 | 0.090 | 2.452 | 0.006 |
| Pond | Climate regulation | 196 | 259 | 594 | 493 | | 350.6 | 457.8 | 499.8 | 525.3 | | 87519.7 | 133715.3 | 244938.7 | 259079.1 | | 4 | 1542 | 481.9 | 213783.6 | 0.014$^{***}$ | 7.333 | 0.057 | 2.660 | 0.000 |
| | Biodiversity maintenance | 907 | 1358 | 681 | 69 | | 1644.5 | 2308.2 | 1418.8 | 470.1 | | 3200551.7 | 4748439.1 | 2467639.3 | 143007.4 | | 4 | 3015 | 1865.6 | 3854831.3 | 0.050$^{***}$ | 52.785 | 0.013 | 2.619 | 0.000 |
| | Environmental purification | 9 | 124 | 356 | | | 1938.8 | 6090.9 | 15728.5 | | | 5172165.5 | 15134111.6 | 195011125.0 | | | 3 | 489 | 13030.8 | 165679433.5 | 0.119$^{***}$ | 32.935 | 0.046 | 3.084 | 0.000 |
| | Water supply | 39 | 63 | 43 | | | 791.8 | 2125.2 | 1788.0 | | | 442639.7 | 4229198.0 | 3066218.0 | | | 3 | 145 | 1666.6 | 3167450.3 | 0.095$^{***}$ | 7.472 | 0.184 | 3.335 | 0.000 |
| Inland mudflat | Biodiversity maintenance | 2 | 16 | 16 | 8 | 15 | 218.5 | 7682.0 | 28512.9 | 29687.9 | 2887.9 | 1308.1 | 85064377.2 | 1428166942.0 | 1476946408.4 | 7618877.7 | 5 | 57 | 15094.4 | 776892984.5 | 0.184$^{*}$ | 2.928 | 0.964 | 2.535 | 0.063 |
| | Aesthetic landscape | 3 | 6 | | | | 12247.8 | 32267.2 | | | | 8073133.6 | 275116738.7 | | | | 2 | 9 | 25594.1 | 275163761.9 | 0.324 | 3.350 | 0.271 | 7.059 | 0.145 |
| Other grasslands | Biodiversity maintenance | 9 | 1 | | | | 3269.8 | 328.1 | | | | 6569562.2 | 0.0 | | | | 2 | 10 | 2975.6 | 6691458.1 | 0.116 | 1.054 | 0.078 | 5.729 | 0.350 |
| | Soil conservation | 25 | 24 | | | | 5356.2 | 2804.1 | | | | 49271242.0 | 7107635.7 | | | | 2 | 49 | 4106.2 | 30247188.4 | 0.054 | 2.673 | 0.101 | 3.100 | 0.126 |
| Sparsely forested woodland | Raw materials production | 148 | 205 | 431 | 360 | 105 | 10441.2 | 6576.6 | 6280.2 | 5648.2 | 9367.7 | 378972436.3 | 137568306.1 | 176377104.8 | 100168414.8 | 300776327.3 | 5 | 1249 | 6899.3 | 185105266.0 | 0.014$^{**}$ | 4.429 | 0.351 | 2.583 | 0.003 |
| | Climate regulation | 98 | 280 | 160 | | | 7566.0 | 11084.8 | 9422.3 | | | 415464438.2 | 915207445.3 | 168557277.5 | | | 3 | 538 | 9949.4 | 603912384.1 | 0.003 | 0.794 | 0.002 | 3.015 | 0.453 |
| | Soil conservation | 6 | 10 | 17 | | | 740.6 | 407.2 | 2165.7 | | | 564950.8 | 77417.4 | 4545975.5 | | | 3 | 33 | 1373.7 | 3147071.0 | 0.216$^{**}$ | 4.127 | 0.324 | 3.833 | 0.040 |
| Other orchards | Raw materials production | 23 | 14 | 11 | | | 867.6 | 735.0 | 935.1 | | | 431537.9 | 120209.7 | 480921.5 | | | 3 | 48 | 844.4 | 357684.8 | 0.016 | 0.360 | 0.221 | 3.549 | 0.725 |
| | Agricultural products supply | 261 | 306 | 444 | 3610 | | 1223.8 | 1248.2 | 1329.6 | 3010.9 | | 1792885.6 | 2704993.4 | 1518.6 | 46501063.2 | | 4 | 4621 | 2631.7 | 37343314.9 | 0.014$^{***}$ | 21.477 | 0.008 | 2.614 | 0.000 |
| | Climate regulation | 44 | 133 | 259 | | | 817.6 | 764.2 | 969.9 | | | 367632.3 | 451131.8 | 904269.2 | | | 3 | 436 | 891.8 | 721032.2 | 0.013 | 2.782 | 0.208 | 3.322 | 0.081 |
| | Soil conservation | 4 | 3 | 30 | | | 609.5 | 334.4 | 821.4 | | | 49666.2 | 17178.7 | 481551.6 | | | 3 | 37 | 759.0 | 417400.1 | 0.048 | 0.864 | 0.207 | 3.606 | 0.465 |
| Irrigated cropland | Raw materials production | 419 | 209 | 75 | | | 500.4 | 615.3 | 724.5 | | | 168620.4 | 239750.0 | 355346.4 | | | 3 | 703 | 558.5 | 215600.5 | 0.027$^{***}$ | 9.869 | 1.091 | 4.448 | 0.000 |
| | Agricultural products supply | 2030 | 2714 | 3250 | 3968 | 18038 | 788.4 | 1042.7 | 1222.2 | 1387.1 | 3633.5 | 488197.6 | 1163312.3 | 2110323.9 | 2577880.1 | 44761549.0 | 5 | 30000 | 2648.2 | 29103101.7 | 0.051$^{***}$ | 402.232 | 0.011 | 2.379 | 0.000 |
| | Climate regulation | 159 | 625 | 1452 | 427 | | 449.9 | 559.6 | 502.8 | 459.0 | | 106746.9 | 234224.8 | 152285.4 | 129759.5 | | 4 | 2663 | 505.9 | 166409.2 | 0.007$^{***}$ | 6.566 | 0.617 | 3.116 | 0.000 |
| | Soil conservation | 35 | 43 | 128 | | | 576.1 | 564.5 | 603.1 | | | 301903.8 | 209648.4 | 287772.2 | | | 3 | 206 | 590.5 | 274140.9 | 0.001 | 0.102 | 0.262 | 3.426 | 0.914 |
| Paddy field | Agricultural products supply | 996 | 1337 | 1864 | 2393 | 23410 | 1295.1 | 1552.5 | 1879.9 | 2121.5 | 4514.2 | 1202127.2 | 1684223.8 | 3195565.9 | 3946233.9 | 36185979.9 | 5 | 30000 | 3920.8 | 30136141.2 | 0.042$^{***}$ | 330.110 | 0.031 | 2.390 | 0.000 |
| | Climate regulation | 362 | 693 | 1091 | 1020 | | 791.2 | 768.2 | 918.9 | 837.8 | | 448285.2 | 459394.2 | 517823.0 | 540648.6 | | 4 | 3166 | 845.2 | 507956.2 | 0.007$^{***}$ | 7.354 | 0.131 | 2.720 | 0.000 |
| Woodland | Raw materials production | 10 | 42 | 90 | 246 | 269 | 1736.4 | 21098.8 | 2359.6 | 22208.9 | 25936.1 | 2429048.5 | 15674686078.1 | 8152968.8 | 1805829883.3 | 3513689142.3 | 5 | 657 | 20633.3 | 3181613064.6 | 0.020$^{**}$ | 3.327 | 0.088 | 2.438 | 0.012 |
| | Climate regulation | 2605 | 4920 | 3990 | 3007 | 3296 | 35714.9 | 44600.8 | 28010.3 | 16506.4 | 12089.5 | 52953064314.5 | 39384176374.0 | 8743311165.6 | 2218641465.9 | 1166336644.0 | 5 | 17818 | 28831.3 | 21318027410.3 | 0.007$^{***}$ | 32.240 | 0.027 | 2.389 | 0.000 |
| | Biodiversity maintenance | 99 | 30 | 6 | 64 | 312 | 634.4 | 797.9 | 870.1 | 3609.3 | 4782.0 | 451087.0 | 321425.8 | 289004.5 | 13762302.1 | 180714345.6 | 5 | 511 | 3551.7 | 115274589.6 | 0.027$^{**}$ | 3.499 | 0.057 | 2.423 | 0.009 |
| | Soil conservation | 16 | 157 | | | | 1820.4 | 2956.7 | | | | 2614387.1 | 48125228.9 | | | | 2 | 173 | 2851.6 | 44024504.6 | 0.002 | 0.422 | 0.016 | 3.958 | 0.520 |

Note: "$N_1, N_2, \cdots, N_5, \mu_1, \mu_2, \cdots, \mu_5$, $\sigma1, \sigma2, \cdots, \sigma5$, $L$, $N$, $\bar{Y}$, $\sigma^2$, $q$-value, $F$, $\lambda$, $F_\alpha$, $P$-value" have the meanings stated in Table S2.
